# ATRX regulates glial identity and the tumor microenvironment in IDH-mutant glioma

**DOI:** 10.1186/s13059-021-02535-4

**Published:** 2021-11-11

**Authors:** Husam Babikir, Lin Wang, Karin Shamardani, Francisca Catalan, Sweta Sudhir, Manish K. Aghi, David R. Raleigh, Joanna J. Phillips, Aaron A. Diaz

**Affiliations:** Department of Neurological Surgery, University of California, Aaron Diaz, 1450 3rd Street, San Francisco, CA 94158 USA

## Abstract

**Background:**

Recent single-cell transcriptomic studies report that IDH-mutant gliomas share a common hierarchy of cellular phenotypes, independent of genetic subtype. However, the genetic differences between IDH-mutant glioma subtypes are prognostic, predictive of response to chemotherapy, and correlate with distinct tumor microenvironments.

**Results:**

To reconcile these findings, we profile 22 human IDH-mutant gliomas using scATAC-seq and scRNA-seq. We determine the cell-type-specific differences in transcription factor expression and associated regulatory grammars between IDH-mutant glioma subtypes. We find that while IDH-mutant gliomas do share a common distribution of cell types, there are significant differences in the expression and targeting of transcription factors that regulate glial identity and cytokine elaboration. We knock out the chromatin remodeler ATRX, which suffers loss-of-function alterations in most IDH-mutant astrocytomas, in an IDH-mutant immunocompetent intracranial murine model. We find that both human ATRX-mutant gliomas and murine ATRX-knockout gliomas are more heavily infiltrated by immunosuppressive monocytic-lineage cells derived from circulation than ATRX-intact gliomas, in an IDH-mutant background. ATRX knockout in murine glioma recapitulates gene expression and open chromatin signatures that are specific to human ATRX-mutant astrocytomas, including drivers of astrocytic lineage and immune-cell chemotaxis. Through single-cell cleavage under targets and tagmentation assays and meta-analysis of public data, we show that ATRX loss leads to a global depletion in CCCTC-binding factor association with DNA, gene dysregulation along associated chromatin loops, and protection from therapy-induced senescence.

**Conclusions:**

These studies explain how IDH-mutant gliomas from different subtypes maintain distinct phenotypes and tumor microenvironments despite a common lineage hierarchy.

**Supplementary Information:**

The online version contains supplementary material available at 10.1186/s13059-021-02535-4.

## Background

Mutations in isocitrate dehydrogenase (IDH) 1/2 define a glioma subtype with a superior prognosis and distinct ontogeny compared to IDH-wildtype glioma [1]. Single-cell and single-nucleus RNA sequencing (sc/snRNA-seq) and in silico lineage tracing of human IDH-mutant gliomas demonstrate a single hierarchy of cellular phenotypes [2], with a neural stem cell (NSC)-like population at the apex giving rise to cells with astrocytic (AC) and oligodendrocytic (OC) expression signatures [3]. Remarkably, this hierarchy is found in all IDH-mutant gliomas regardless of molecular subtype. This finding is somewhat paradoxical because the genetic differences that define IDH-mutant glioma subtypes are prognostic [1], predictive of response to chemotherapy [4, 5], and correlate with distinct tumor microenvironments [2, 6].

Grade II/III IDH-mutant gliomas are classified in the World Health Organization 2016 standard by the presence of a 1p/19q co-deletion as oligodendroglioma (IDH-O) or as 1p/19q-intact astrocytoma (IDH-A) [7]. IDH-A is characterized by a nearly ubiquitous loss of function in ATRX (inactivated in 86% of IDH-A). ATRX mutations are rare in IDH-O. How genetic differences contribute to the differences in tumor histology and microenvironment observed across IDH-mutant glioma subtypes is not known.

We profiled specimens from 22 IDH-A/O untreated human gliomas via single-cell assay for transposase-accessible chromatin (scATAC-seq) and sc/snRNA-seq. These data resolve the cell-type-specific differences between IDH-A and IDH-O in transcription-factor utilization, associated targeting and cis-regulatory grammars. Having mapped open chromatin at cellular resolution, we can confirm a tripartite distribution of cell types common to both IDH-A/O.

However, we also find significant differences in transcription-factor expression and targeting between IDH-A and IDH-O cells of the same type. Proliferating IDH-A cells exploit a gliogenic switch driven by Nuclear factor I (NFI) transcription factors, they upregulate nuclear factor kappa-light-chain-enhancer of activated B cells (NFKB) pathway genes and downstream cytokine expression. This coincides with a higher degree of myeloid-derived cells in IDH-A specimens with an immunosuppressive phenotype. By contrast, cycling IDH-O cells instead upregulate basic helix-loop-helix (bHLH) transcription factors governing OC lineage commitment.

ATRX is a chromatin remodeler which regulates gene expression by modulating nucleosome positioning, chromatin looping, and via other mechanisms [8]. We hypothesized that some of the differences in gene expression and open chromatin observed between IDH-A and IDH-O specimens were mediated by ATRX loss-of-function. Moreover, we reasoned that some of these differences would be conserved and regulated by ATRX in murine gliomas as well. To test this, we knocked out ATRX in an immunocompetent intracranial murine model of IDH-mutant glioma [9]. We subjected knockout and wildtype tumors to scRNA-seq and snATAC-seq and compared them to human tumors.

We found (1) ATRX-deficient, IDH-mutant human and murine gliomas both upregulate a pro-AC regulatory program driven by NFI genes and downregulate a pro-OC program driven by bHLH transcription factors; (2) both human and mouse ATRX-deficient, IDH-mutant gliomas upregulate genes that promote myeloid-cell chemotaxis and both have significantly higher percentages of myeloid-derived cells expressing immunosuppressive factors than controls; (3) a transcription-factor program is conserved between human and murine ATRX-deficient tumors that drives an AC phenotype and cytokine elaboration; and (4) ATRX loss protects human and murine glioma cells from therapy-induced senescence.

We conclude that ATRX loss-of-function in IDH-mutant glioma orchestrates chromatin and gene-expression differences that regulate glial identity and myeloid-cell induction. These studies shed light on the genetic regulation of histology and microenvironment composition in IDH-mutant glioma.

## Results

### A single-cell multi-omics atlas of IDH-mutant glioma identifies a tripartite distribution of cell types

**Fig. 1:**
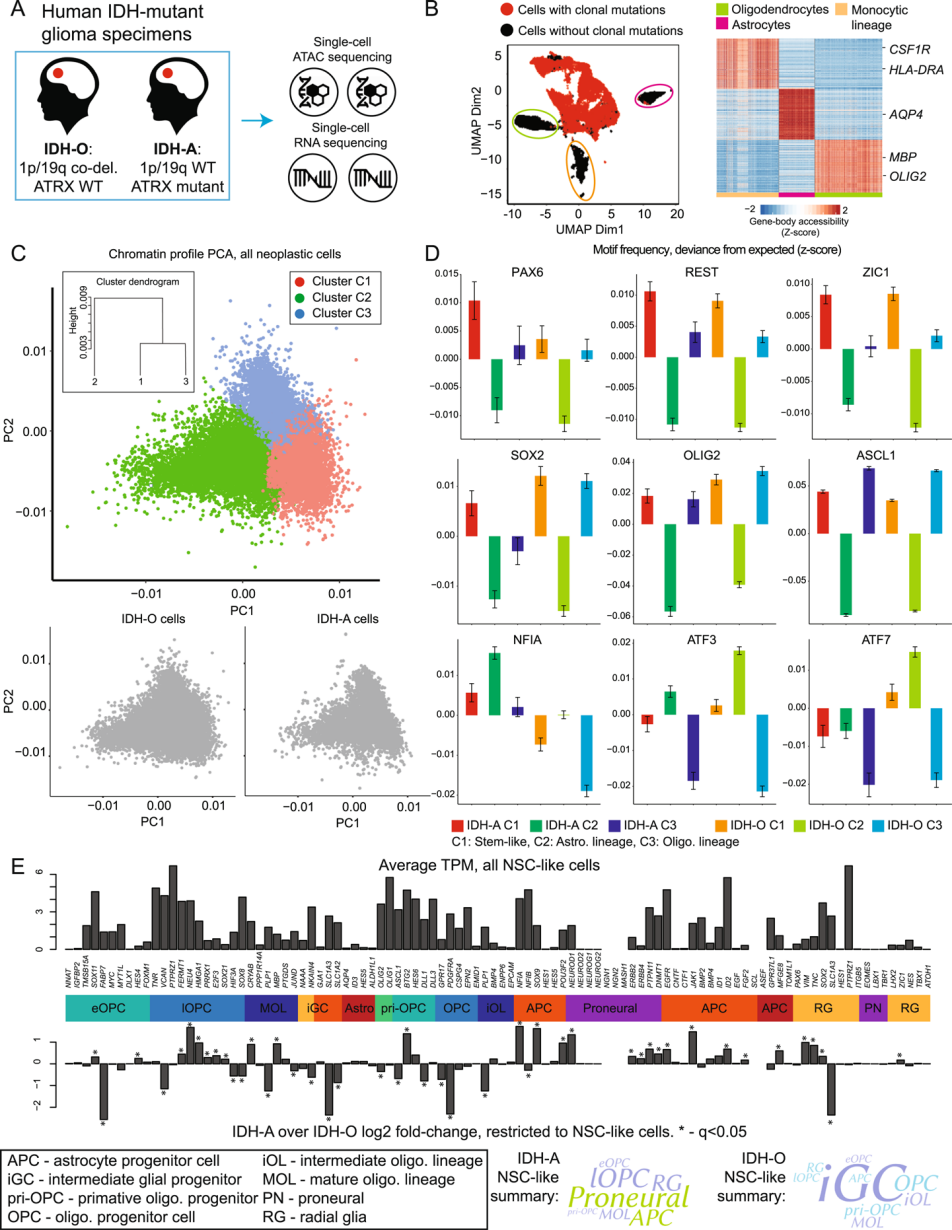
A single-cell chromatin atlas of IDH-mutant glioma. **A** Overview of the analysis of human clinical specimens. **B** Summary of the scATAC-seq data and separation of neoplastic cells. **C** PCA and clustering of neoplastic cells from the scATAC-seq data, based on genome-wide open-chromatin signatures. The first two PCA components are hierarchically clustered via Pearson correlation as a metric and Ward’s linkage. **D** Transcription-factor motif frequencies in scATAC-seq data for select motifs that are differentially enriched between IDH-A and IDH-O neoplastic cells at *q*<0.05 (Table 3), assessed via ChromVAR, represented as standardized deviances from expected (Methods), and separated by cluster. Error bars represent standard error. **E** Average expression (top) and log_2_ fold-change between IDH-A and IDH-O (bottom) for stem-like neoplastic cells, restricted to gene sets previously implicated during neuronal and glial genesis, assessed via limma (Additional file 3: Table S2 and Additional file 4: Table S3). The percentages of each cell-type’s gene set that are differentially expressed in IDH-A vs. IDH-O stem-like cells are represented as word clouds below. The higher the percentage, the larger the word

We profiled 22 human IDH-mutant grade II/III gliomas via snATAC-seq: 10 ATRX-mutant IDH-A and 12 ATRX wildtype, 1p/19q co-deleted IDH-O tumors (Fig. 1A, Additional file 1: Fig. S1A-C; Additional file 2: Table S1; Methods). This generated open-chromatin profiles for 38,552 cells following quality control. We separated neoplastic from non-neoplastic cells and classified cell types using our previously described approaches [3, 10, 11]. This identified neoplastic cells harboring clonal mutations, unmutated glia, and monocytic-lineage cells (Fig. 1B).

Having isolated neoplastic cells, we used a SnapATAC-based [12] pipeline to bin reads and cluster cells (Methods). We identified three populations, which we visualized using a principal components analysis (PCA; Fig. 1C). The three clusters were stratified by the first two principal components, indicating that inter-cluster differences represent the primary source of variation in these data. Notably, the percentages of cells in each cluster were nearly equal between IDH-A and IDH-O specimens (Additional file 1: Fig. S1D).

To interpret these clusters, we used ChromVAR to scan sequenced reads from each cluster and identify sequences matching known transcription-factor motifs. We identified motifs that were significantly over- or under-represented compared to a data-driven background distribution. While both cluster one and cluster three were enriched for transcription factors associated with glioma-propagating cells (e.g., SOX2), markers of oligodendrocyte-progenitor cells (OPCs) were more enriched in cluster three (Fig. 1D, Additional file 1: Fig. S1E-F). Cluster one alone showed over-representation of motifs from proneural transcription factors that are expressed by NSCs in the ventricular and sub-ventricular zones of the developing human neocortex (e.g., ZIC1; Additional file 1: Fig. S1G). Cluster 2 was characterized by a depletion of makers of NSCs or OC lineage. Rather, the motifs of AC-lineage regulators (e.g., NFIA, ATF3) [13] were abundant in cluster two. These data confirm a tripartite distribution of cell types (AC, OC, and NSC-like) observed previously by ourselves and others [3, 14], while additionally resolving the transcription-factor programs that mediate differences between these phenotypes.

We profiled seven IDH-mutant grade II/III gliomas via scRNA-seq (Additional file 2: Table S1). We combined this with scRNA-seq data from 13 published IDH-mutant grade II/III gliomas. The combined cohort was comprised of both ATRX-mutant 1p/19q intact IDH-As and ATRX-wildtype 1p/19q-codeleted IDH-Os [14]. Quality control and separation of neoplastic cells from immune cells, nonmalignant glia, and endothelium were performed via our previously described approaches [10, 11, 15]. We further classified neoplastic cells into AC, OC, and NSC-like populations based on the signatures and procedure of Venticher et al. [14]. Cell-type-specific gene expression largely correlated with cluster-specific differences in motif frequency observed in the scATAC-seq data (Additional file 3: Table S2).

### Proliferating IDH-A and IDH-O neoplastic cells utilize distinct glial-lineage programs

We compared gene expression in the NSC-like populations between IDH-A/O and to recent scRNA-seq-based lineage tracing studies performed in human and murine fetal brain tissues (Fig. 1E, Additional file 4: Table S3). This included studies mapping the transition from radial glia (a multipotent progenitor responsible for producing both cortical neuron and glial progenitors) to neuronal, astrocyte, and oligodendrocyte progenitors [16–19], as well as transitions along the oligodendrocyte [20] and astrocyte [21–23] lineages from glial progenitors to terminally differentiated glia. We found that NSC-like cells from both IDH-A/O gliomas express markers common to radial glia and glioblasts, while downregulating markers of mature AC and OC cells. However, proneural and pro-astrocytic transcription factors (e.g., *NFIA*) are upregulated in IDH-A NSC-like cells. Most of the markers that are significantly upregulated in IDH-O NSC-like cells were OC-lineage markers.

**Fig. 2:**
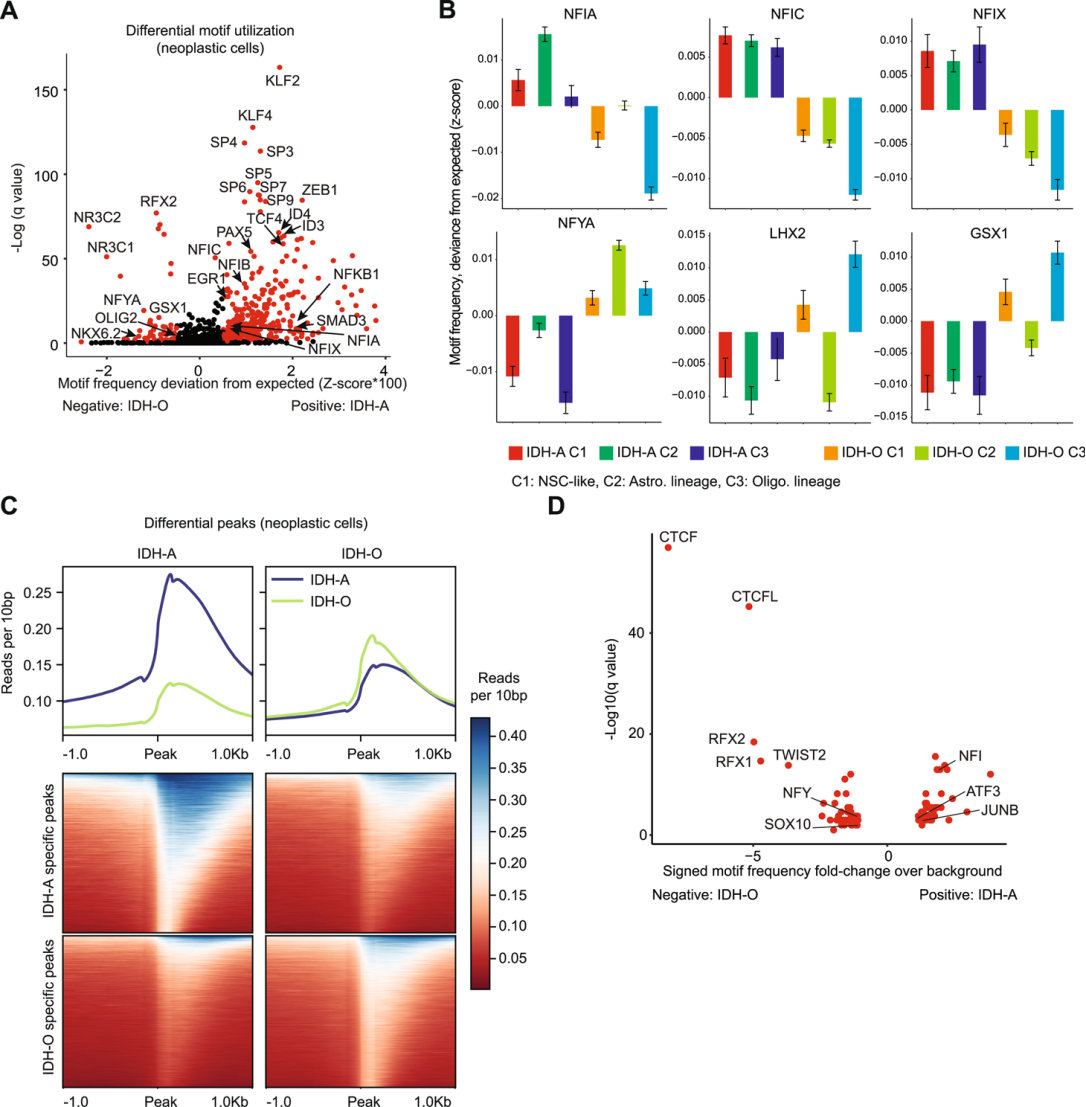
Differences in open chromatin and transcription-factor targeting between IDH-A and IDH-O. **A** Differential test for motif frequency in the scATAC-seq data between IDH-A and IDH-O neoplastic cells via ChromVAR. **B** Transcription-factor motif frequencies in scATAC-seq data for selected motifs that are differentially enriched between IDH-A and IDH-O neoplastic cells at *q*<0.05 (Table 3), assessed via ChromVAR, represented as standardized deviances from expected (Methods), and separated by cluster. Error bars represent standard error. **C** Differential peaks from scATAC-seq data called between IDH-A and IDH-O neoplastic cells via MACS at *p*<0.05. Peaks are represented as a heatmap (bottom) and a moving average (top) of reads per 10 bp, in a 2 Kbp window around the transposase cut site. **D** Over-represented transcription-factor motifs in differential peaks from scATAC-seq data, comparing IDH-A and IDH-O neoplastic cells. Over-representation compared to a background distribution was performed via HOMER, only motifs with *q*<0.05 are shown

There are also significant differences in both the expression and motif enrichment for NFI, bHLH, and other transcription factors that we found when comparing IDH-A and IDH-O cells of the same cell type. We performed a differential test of transcription-factor motif frequencies between IDH-A and IDH-O cells (Fig. 2A, B, Additional file 1: Fig. S2A-B, Additional file 5: Table S4; Methods). We found significant increases of NFI transcription-factor expression and targeting in IDH-A cells, particularly in the cycling, NSC-like population (Figs. 1E and 2B). NFI gene expression regulates glia-genesis and NFIA in particular mediates AC-lineage commitment [22]. Additionally, we saw increases in Notch pathway (e.g., JAG1) and mitogen-associated pathway (MAP) kinase pathway genes in IDH-A cells. Conversely, IDH-O cells upregulate expression and motif frequency of genes governing OC lineage [20].

We called peaks in the scATAC-seq data and identified regions of differentially accessible chromatin between IDH-A and IDH-O specimens. Although we did identify differential peaks in both IDH-O and IDH-A datasets, IDH-A neoplastic cells were characterized by a marked increase in open chromatin compared to IDH-O cells (Fig. 2C). Motif analysis of differential peaks (Fig. 2D, Additional file 1: Fig. S2C) identified CCCTC-binding factor (CTCF), and paralog CTCF-like (CTCFL, aka BORIS), as the two most significantly over-represented in IDH-O cells. ATRX deficiency leads to changes in nucleosome density at CCCTC-motif sequences, reduced CTCF binding, changes in chromatin looping and gene expression in the neonatal brain [24]. ATRX-mutant colorectal cancer cells show reduced ability to form heterochromatic foci compared to wildtype controls [25]. This is consistent with the fact that all our IDH-A specimens (and over 86% of the IDH-A patient population) are ATRX mutant while the IDH-O samples (and most IDH-O gliomas) have ATRX intact. This led us to hypothesize that at some of the chromatin differences we observed between IDH-A and IDH-O tumors are due to ATRX loss of function.

### IDH-As are more heavily infiltrated by monocytic-lineage cells derived from circulation, they upregulate myeloid-cell chemotaxis genes and upstream transcription factors, compared to IDH-Os

**Fig. 3:**
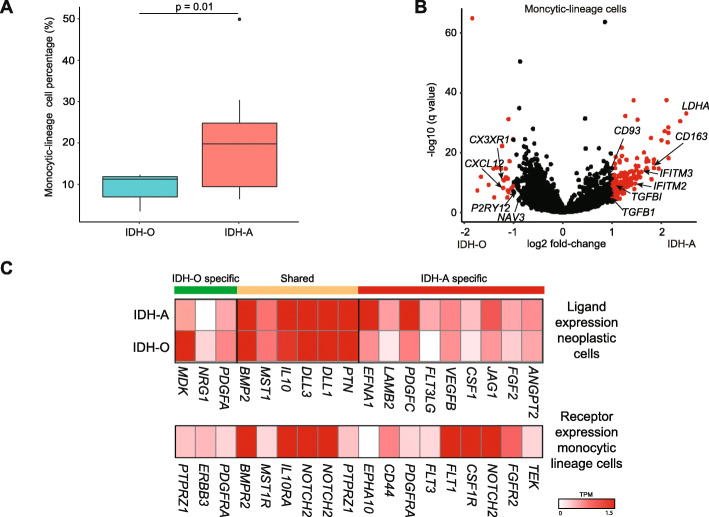
Differences in immune-cell phenotypes, paracrine signals, and upstream transcription factors between IDH-A and IDH-O*.* **A** Percentages of monocytic-lineage cells found in IDH-A vs. IDH-O scRNA-seq data, and t-test. **B** Differential expression test via MAST for monocytic-lineage cells from scRNA-seq of IDH-A and IDH-O specimens. **C** Ligand/agonist expression in neoplastic-cell scRNA-seq from IDH-A and IDH-O specimens (top) paired to expression of the corresponding receptors in monocytic-lineage cells (bottom)

We found significantly higher percentages of monocytic-lineage cells in IDH-A tumors compared to IDH-O (Fig. 3A). The majority of monocytic-lineage cells from IDH-As were classified as macrophages derived from the peripheral blood, according to our previously described markers [6] and approach [11], while IDH-O monocytic-lineage cells were mostly microglia. Monocytic-lineage cells from IDH-A specimens over-expressed markers of a tumor-supportive phenotype (e.g., CD163; Fig. 3B), compared to IDH-O specimens. IDH-A monocytic-lineage cells also highly over-expressed lactate dehydrogenase relative to IDH-O samples (Fig. 3B), a hallmark of “M2” glycolytic metabolism that is implicated in enhancing macrophage extravasation of the blood-brain barrier [26] and in local immunosuppression [27]. Thus, IDH-A specimens are skewed toward greater infiltration of monocytic-lineage cells, expressing markers of bone marrow ontogeny and M2 polarization.

To elucidate differences in myeloid-neoplastic paracrine signaling between IDH-A and IDH-O, we identified ligands expressed by neoplastic cells whose cognate receptors were also expressed by monocytic lineage cells (Fig. 3C). Notably, myeloid chemotaxis factors (e.g., *CSF1*, *FLT3LG*) are over-expressed in IDH-A, as is the upstream transcription factor *NFKB1*. The latter correlates with significant increases in NFKB1 motif frequency (Additional file 1: Fig. S2A). Likewise, IDH-A specimens show correlated motif-usage and expression increases in ETS1 and increased *FYN* expression (Additional file 1: Fig. S2A, Additional file 3: Table S2), all regulators of inflammatory cytokines. We mapped differential scATAC-seq peaks called between IDH-A and IDH-O neoplastic cells to adjacent genes and correlated the results with snRNA-seq expression data (Additional file 1: Fig. S3A-B). We compared these correlated peaks to databases of transcription-factor binding sites (Additional file 1: Fig. S3C; Methods). We found an enrichment for the reported binding sites of ATRX co-factor DAXX within peaks correlating with lDH-A-specific ligand expression. This further supported our hypothesis that some of the observed differences between IDH-A and IDH-O microenvironments are mediated by ATRX loss of function.

### An immunocompetent mouse model of ATRX-deficient glioma

**Fig. 4:**
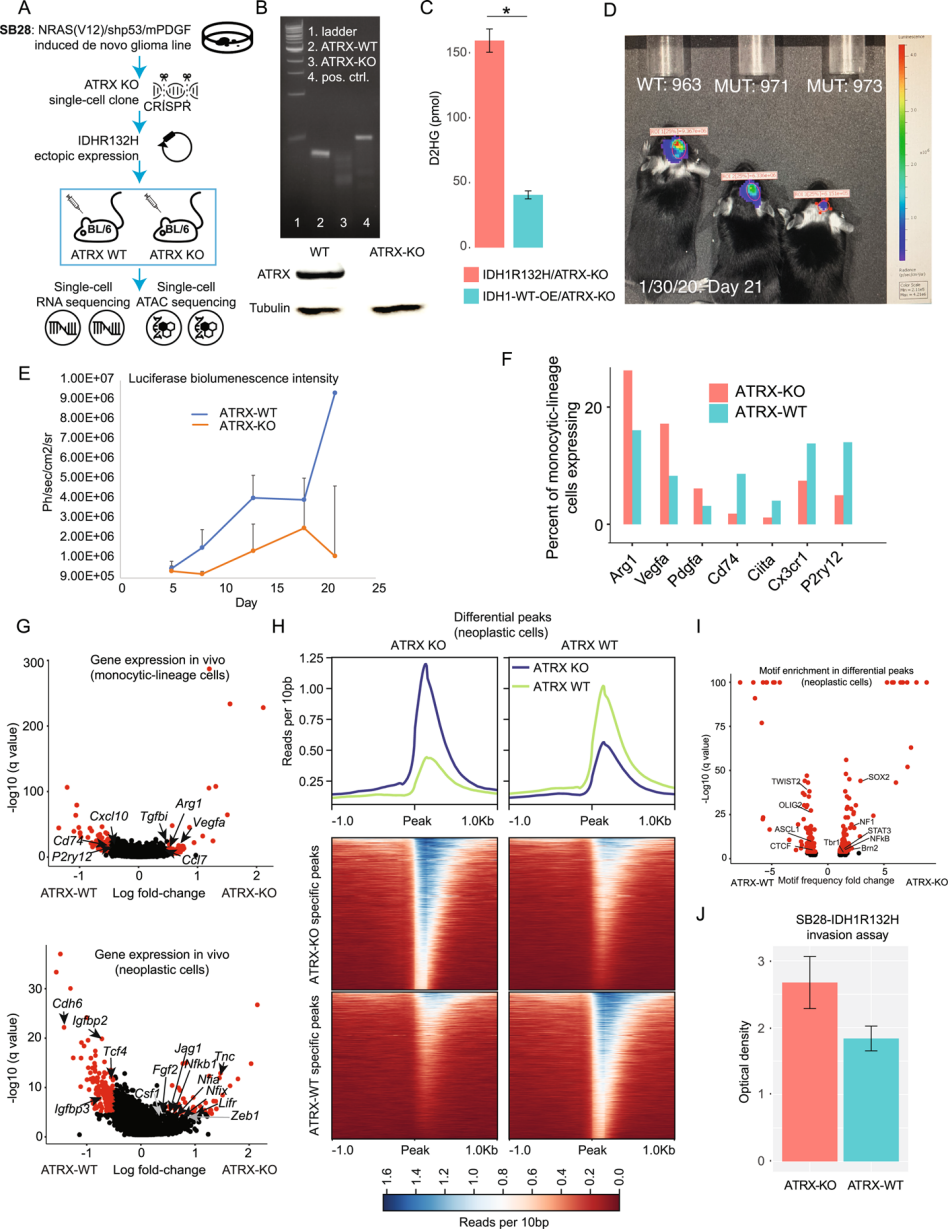
Phenotypic differences between ATRX knockout and wildtype gliomas in an IDH-mutant background. **A** A schematic overview of the murine studies. **B** An enzymatic cleavage assay, indicating homozygous knockout in ATRX exon 9 (Top), a Western blot showing complete loss of ATRX protein (bottom). **C** Fluorometric 2-HG detection assay, comparing wildtype IDH1 and IDH1R132H overexpression in ATRX-KO SB28 cells, *t test *p*<0.05. **D** Representative BLI images of ATRX-KO and wildtype intercranial tumors. **E** BLI timeseries. **F** Percentages of tumor-infiltrating monocytic-lineage cells expressing the given markers in ATRX-KO vs. wildtype, in an IDH1R132H background. **G** (Top) Differential expression test of snRNA-seq data via MAST, comparing tumor-infiltrating monocytic-lineage cells between ATRX-KO and wildtype tumors in an IDH1R132H background. (Bottom) Differential expression test of snRNA-seq data via MAST, comparing neoplastic cells between ATRX-KO and ATRX-wildtype tumors in an IDH1R132H background. **H** Differential peaks from scATAC-seq data called between neoplastic cells from ATRX-KO and ATRX-wildtype tumors with an IDH1R132H background. Peaks are called via MACS at *p*<0.05 and represented as a heatmap (bottom) and moving average (top) of reads per 10 bp, in a 2 Kbp window around the transposase cut site. **I** ScATAC-seq motif enrichment in differential peaks between ATRX-KO and ATRX-wildtype neoplastic cells. Motif frequency relative to a genome-wide background was scored via HOMER. **J**) Extracellular-matrix invasion assay, comparing ATRX-KO and wildtype cells with an IDH1R132H background

To model ATRX deficiency in IDH-mutant glioma, we used the immunocompetent glioma model of SB28 cells injected intracranially in C57BL/6j mice as a starting point (Fig. 4A) [9, 28]. These cells produce a relatively modest immune response when injected into the mouse brain.

We transfected SB28 cells with plasmids expressing CRISPR double-nickase guide-RNAs targeting exon 9 of Atrx, as well as Cas9 nuclease (Methods). The CRISPR double-nickase platform uses two guide RNAs which target proximal sequences. The colocalization of both guide-RNAs is required to generate durable DNA alterations. This system, therefore, has much lower off-target rates than single-guide CRISPR systems. Following selection, we isolated single live cells via flow cytometry and expanded them as clones. We then screened clones for a homozygous ATRX knockout (KO) via a cleavage assay and validated the complete loss of ATRX protein via Western blot (Fig. 4B). We then transfected ATRX-KO and wildtype (WT) cells with plasmids expressing the most common IDH1 mutant, IDH1R132, or an IDH-wildtype control. Following selection, we confirmed over 3-fold increase in 2-hydroxyglutarate (the metabolic effector of mutant IDH) in transformed cells compared to controls (Fig. 4C) via an enzymatic assay [29].

### ATRX-KO murine gliomas are more heavily infiltrated by immunosuppressive blood-derived monocytic lineage cells than controls, in an IDH-mutant background.

We performed intracerebral injections of ATRX-KO/IDH1R132H or ATRX-WT/IDH1R132H SB28 cells into the right cerebral hemispheres of mice, 50,000 cells per mouse, 6 mice per cohort. We monitored BLI intensity bi-weekly and found that both ATRX KOs and controls generated robust signals in all mice (Fig. 4D, E). Mice were sacrificed when they showed hunchback, seizures, hemiparesis, or weight loss of greater than 20%; two control mice and one ATRX-KO mouse were found dead and thus excluded.

Mice were perfused, and tumor tissue was harvested and snap-frozen. We then performed snRNA-seq and scATAC-seq on a cohort of tumors from five ATRX-KO and four wildtype mice, pooling tissue across ATRX-KO and control mice, respectively. We separated neoplastic cells and classified tumor-infiltrating cells based on our previously described approaches [6, 10]. We found significant increases in the percent of tumor-infiltrating macrophages derived from the peripheral blood compared to microglia (Fig. 4F). Tumor-infiltrating monocytic-lineage cells from ATRX KOs expressed less class II HLA and higher levels of *Arg1*, *Vegfa*, and other markers of the immunosuppressive M2 phenotype (Fig. 4F, G, Additional file 6: Table S5). Thus, the myeloid-rich, inflammatory microenvironment of the ATRX-KO/IDH1R132H SB28 model parallels that of ATRX-mutant IDH-A.

### ATRX loss-of-function enhances glioma invasiveness, pro-AC, and pro-inflammatory regulatory programs

We found changes in gene expression and correlated changes in transcription-factor motif frequencies in neoplastic cells isolated from ATRX-KO murine tumors, compared to wildtype controls. Moreover, many of these differences agreed with the differences observed between IDH-A and IDH-O human tumors. These differences were organized around three pathways: genes regulating glial linage identity, genes regulating cytokine secretion, and genes enhancing invasiveness.

In particular, ATRX KO induces NFI transcription factors *Nfia* and *Nfix* in vivo (Fig. 4G, Additional file 6: Table S5), both regulators of the AC-lineage [13, 21, 22]. This correlates with an over-representation of NFI motifs in the scATAC-seq peaks specific to ATRX-KO specimens, but not observed in wildtype controls (Fig. 4H, I, Additional file 1: Fig. S4A, Additional file 7: Table S6). Thus, the correlation between increased NFI gene expression and motif usage that we had observed in human ATRX-mutant gliomas (Figs. 1 and 2) is conserved in ATRX-KO murine tumors. Likewise, we saw enrichment for OLIG2 and ASCL1 motifs in ATRX wildtype human and ATRX-WT specimens.

We found that in both ATRX KO and WT cells scATAC-seq open-chromatin signatures in neoplastic cells were divided into three clusters. The first was depleted of the stemness and proliferation TF motifs SOX2 and JUNB and dominated by TF motifs of core components of the expression and transduction of pro-inflammatory cytokines, e.g., NFKB and STAT3 signaling (Additional file 1: Fig. S4C-D). The second and third were more closely aligned with each other, enriched for SOX2 and JUNB but differed in the relative frequencies of glial-lineage TF motifs. We found a relatively homogeneous shift in all populations in the frequencies of glial lineage motifs from oligodendrocyte-lineage markers (e.g., OLIG2) to astrocytic (e.g., NFIA) across all three populations. The impact of ATRX deficiency on the global epigenome appears to be relatively homogeneous in our model.

The master-regulator of inflammation, *Nfkb1,* shows significantly upregulated gene expression in ATRX-KO and its recognition motif is enriched in ATRX-KO-specific peaks. This was similarly the case for human ATRX-mutant gliomas. Similarly, transcription-factor regulators (e.g., ZEB1) and enhancers (e.g., TNC) of invasion were significantly increased both in human ATRX-mutant and ATRX-KO murine glioma specimens. This again correlated with significant differences in motif frequency. We found that ATRX-KO glioma cells were more invasive in vitro than ATRX-wildtype controls, in an IDH1R132H background (Fig. 4J). This dovetails with previous findings that ATRX knockout enhances TNC expression and motility in murine neural stem cells [30]. We conclude that ATRX loss-of-function leads to significant changes in transcription factor expression and targeting. These changes regulate glial identity and cytokine expression programs that phenocopy human IDH-A.

### ATRX loss induces a global decrease in CTCF binding, correlated gene dysregulation, and protection from therapy-induced senescence

**Fig. 5:**
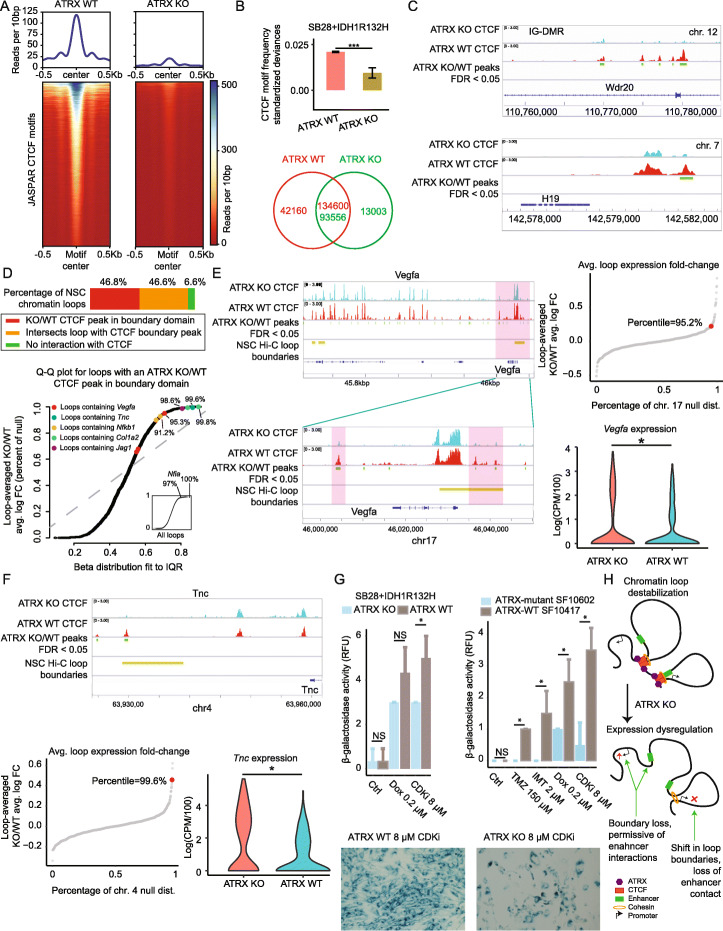
ATRX loss induces a global loss of CTCF, correlated gene dysregulation, and protects glioma cells from therapy-induced senescence. **A** Anti-CTCF single-cell CUT&Tag in SB28+IDH1R132H cells with ATRX KO or ATRX-wildtype scrambled control. Reads per 10 bp are shown as heatmaps (bottom) and summarized as averages (top) in a neighborhood of JASPAR CTCF motif sites. **B** CTCF motif frequencies in scCUT&Tag peaks, represented as standardized deviances from a data-driven null distribution via ChromVAR, for ATRX KO vs. wildtype cells (top), ***t test *p* < 1e−16. Numbers of, and overlap between, peaks in ATRX KO and wildtype datasets. **C** Browser shots of CTCF scCUT&Tag read pile-ups with significant peaks and genes annotated. **D** The percentages of NSC chromatin-loops affected, directly or indirectly, by CTCF loss upon ATRX KO (top). A Q-Q plot of the average ATRX-KO/WT cell-averaged log fold-changes in gene expression for each chromatin loop determined from NSC Hi-C data. Values on the *y*-axis are represented as percentages of a corresponding null distribution (Methods). The *x*-axis quantiles are provided by a Beta distribution fit to the inter-quartile range of *y*-axis percentiles. Loops containing specific genes are annotated. **E** Browser shots of a chromatin loop containing VEGFA and closeup of one boundary domain (left). A null distribution of average ATRX-KO/WT cell-averaged log fold-changes in gene expression measured via snRNA-seq in vivo in neoplastic cells, taken across all consecutive windows of four adjacent genes in chromosome 17. The loop containing VEGFA and the percentile of its average log fold-change are annotated (right-top). Violin plots of *Vegfa* expression in ATRX KO and WT neoplastic cells, in vivo, *MAST *q* < 0.05 (right-bottom). **F** As in **E**, except for a loop containing TNC. **G** Fluorometric assay for β-galactosidase activity in ATRX-KO and scrambled-control SB28 cells, both expressing IDH1R132H, post-treatment with one of Doxorubicin or CDKi. Error bars indicate standard error. *t test *p* < 0.05. **B** As in **A**, but for patient-derived ATRX/IDH1 double-mutant cells (SF10602) compared with an ATRX-wildtype, IDH1-mutant control (SF10417). Cells were treated with one of Temozolomide, Imatinib, Doxorubicin, or CDKi. **C** Visualization of β-galactosidase expression in ATRX-KO and scrambled-control SB28 cells, both expressing IDH1R132H, before and after treatment with CDKi. **H** Model of gene dysregulation due to CTCF disruption in chromatin-loop boundaries

Notably, CTCF recognition motifs were significantly over-represented in the scATAC-seq peaks that were specific to ATRX-wildtype SB28+IDH1R132H cells (*q*=0.0197), this was not the case for peaks specific to ATRX-KO cells (Fig. 4I, Additional file 7: Table S6). This is consistent with our human glioma scATAC-seq data, in which CTCF recognition motifs are the most over-represented in ATRX-wildtype specimens (c.f. Fig. 2D). It has been recently shown that ATRX-mutant colorectal cancer cells are resistant to therapy-induced senescence, due to an inability of ATRX-mutant cells to form the requisite heterochromatic foci [25]. Moreover, ATRX loss-of-function leads to a loss of CTCF binding and chromatin dysregulation in the developing brain [24]. To test if ATRX mediates CTCF binding in IDH-mutant glioma we performed anti-CTCF single-cell Cleavage Under Targets and Tagmentation (scCut&Tag) on ATRX wildtype (scrambled control) and KO SB28+IDH1R132H cells (Methods). We found reads aggregated around CTCF motifs defined via the JASPAR database in ATRX-wildtype cells, likewise in an ATRX-wildtype glioblastoma specimen used as a positive control, but this was not the case in ATRX-KO cells (Fig. 5A, Additional file 1: Fig. S1A-B). Sequenced reads from both ATRX-wildtype and ATRX-KO cells showed a significant over-representation of CTCF motifs compared to a data-driven background distribution, with significantly higher representation in ATRX-wildtype vs. ATRX-KO cells; similarly, a greater number of CTCF peaks were identified via MACS2 at an FDR of 0.05 in ATRX-wildtype vs. ATRX-KO cells (Fig. 5B; Methods). Lastly, as additional controls, we interrogated CTCF binding in imprinted regions that have been previously described [24, 31] as loci of ATRX-mediated CTCF binding and found significant differential CTCF enrichment in ATRX-wildtype over ATRX-KO conditions (Fig. 5C). We conclude that ATRX KO leads to a significant, global decrease in CTCF binding in IDH-mutant glioma. This explains the differences in CTCF motif frequency observed between ATRX-KO and ATRX-wildtype, as well as between IDH-A and IDH-O, IDH-mutant glioma specimens. When we compared KEGG pathway enrichment of genes within 25kbp of ATRX-WT/KO differential peaks to ATRX-KO/WT differentially expressed genes, we found co-enrichment of focal adhesion, Ras and MAPK signaling, proteoglycans, and other pathways (Additional file 1: Fig. S5C), supporting the conclusion that the observed changes in CTCF binding were related to the observed changes in gene expression between ATRX-KO and ATRX-WT neoplastic cells in vivo.

Recent studies support our conclusion that a global loss of CTCF binding explains the global increase in open chromatin observed in ATRX-deficient human and murine gliomas (Fig. 2C and 4H) and point to a mechanism by which these differences regulate gene expression. In particular, Pękowska et al. recently demonstrated that a gain of CTCF anchored chromatin loops was a hallmark of the transition from pluripotency to neuroglial lineage commitment [32]. They mapped CTCF and chromatin conformation genome-wide in murine neural stem cells (NSC) and embryonic stem cells and identified a genome-wide induction of CTCF-anchored loops that accompanied loss of pluripotency in NSCs. We compared their annotation of NSC chromatin loops and associated loop-boundary domains to our CTCF peaks in ATRX-KO and wildtype conditions (Fig. 5D). Consistent with the global depletion of CTCF observed upon ATRX KO, we found that approximately 47% of loops contained an ATRX-WT/KO differential peak in their loop-boundary domain, and another 47% of loops overlapped a CTCF-altered loop. To determine which loops demonstrated a coordinated change in gene expression upon ATRX KO, for each loop, we averaged the ATRX-KO/WT cell-averaged log fold-changes in gene expression from neoplastic cells based on in vivo snRNA-seq. To compare these loop-wide averages to a data-driven background estimate, for each loop, we computed a distribution of comparable averages by considering all stretches of consecutive genes of the same length, along the same chromosome (Methods). We then represented the average log fold-change for each loop as a percentile of its associated distribution. We found coordinated loop-wide changes in gene expression, correlating with differential ATRX-WT/KO CTCF peaks in associated loop-boundary domains, for loops containing Vegfa, Tnc, Nfkb1, and others (Fig. 5D–F, Additional file 1: Fig. S5D-E).

We reasoned that the loss of CTCF binding and global chromatin de-condensation that we observed upon ATRX KO may also explain the inability to form the heterochromatic foci necessary for therapy-induced senescence reported recently in ATRX-deficient cancers [25]. We found that ATRX-KO, IDH1R132H-overexpressing SB28 cells were resistant to therapy-induced senescence, as assayed via β-galactosidase activity (Methods), when treated with doxorubicin or a cyclin-dependent kinase inhibitor (CDKi). Likewise, ATRX-mutant patient-derived glioma cells were found to be more resistant than a wild-type control to senescence induced by Temodar, Imatanib, Doxorubicin, or CDKi treatment, in an IDH1-mutant background (Fig. 5G, Additional file 1: Fig. S5F). We conclude that ATRX loss-of-function leads to a decrease in CTCF binding and associated heterochromatin, enabling human and murine glioma cells to evade the desirable outcome of therapy-induced senescence.

## Discussion

We constructed a single-cell atlas of chromatin structure in human IDH-mutant glioma. An integrated analysis of snRNA-seq and snATAC-seq data confirmed that AC, OC, and NSC-like phenotypes are common to both IDH-A and IDH-O specimens. However, these studies also show that there are significant differences in transcription-factor expression and targeting that distinguish IDH-A and IDH-O. Our murine studies show that many of these differences are due to ATRX loss of function.

NFI genes were enriched, both in terms of higher RNA expression and higher recognition-motif frequency in open chromatin, in IDH-A specimens compared to IDH-O. NFIA is a master-regulator of astrogenesis [13, 21, 22] and is sufficient to impart glial competency to neural stem cells [33]. On the other hand, we found enrichment of transcription factors that regulate the OC-lineage (e.g., OLIG2, ASCL1, *PDGFRA*) in IDH-O specimens. These differences between IDH-A and IDH-O cells were present even when we restricted our analysis to cells of the same phenotype, e.g., stem-like glioma cells (Fig. 1E). We conclude that although IDH-A and IDH-O share a common tripartite distribution of cell types (Fig. 1C), transcription-factor networks governing glial lineage specification diverge (Fig. 1D-E, Additional file 1: Fig. S1B-C, Additional file 3: Table S2, Additional file 5: Table S4). Thus, differences in glial identity observed between IDH-A and IDH-O are more directly explained by differences in transcription-factor expression and chromatin accessibility than by differences in the abundance of AC or OC cells, as the percentages of AC/OC/NSC-like cell types observed in IDH-A and IDH-O specimens are nearly equal (Additional file 1: Fig. S1A).

We found that ATRX knockout in an IDH-mutant background enhanced NFI gene expression in vivo (Fig. 4G, Additional file 6: Table S5). Moreover, NFI recognition motifs were over-represented in the peaks that were specific to ATRX-KO tumors, compared to wildtype controls. Conversely, peaks lost in ATRX-KO tumors were enriched for regulators of the oligodendrocyte lineage observed in IDH-O (e.g., OLIG2, ASCL1; Fig. 4I, Additional file 7: Table S6). Therefore, ATRX-KO IDH1R132H murine glioma phenocopies the glial regulatory program observed in IDH-A.

Another clinically relevant difference between IDH-A and IDH-O is the abundance and phenotype of tumor-infiltrating monocytic-lineage cells. We have shown previously that the accumulation of myeloid-derived cells in IDH-mutant glioma correlates with significantly inferior survival [6]. We now expand on that finding to show that monocytic-lineage cells derived from the peripheral blood more heavily infiltrate IDH-A and ATRX-KO/IDH1R132H gliomas, compared to IDH-O and ATRX-wildtype/IDH1R132H glioma, respectively. We describe the milieu of ligand and agonist expression in IDH-mutant glioma that is relevant for myeloid-neoplastic paracrine signaling (Fig. 3C) and elucidate upstream transcription factors (Additional file 1: Fig. S3). For example, IDH-A tumors upregulate *CSF1* and *FLT3LG* which are chemotactic for monocytic-lineage cells, compared to IDH-Os. These findings show how IDH-A and IDH-O maintain distinct tumor microenvironments, despite having a similar composition of neoplastic glial cell types.

Pan-cancer analysis has identified a T cell-poor, macrophage-rich, phenotype that is found in solid tumors and which correlates with ATRX mutations across malignancies and specimens [34]. We found that ATRX knockout in IDH-mutant glioma led to increased infiltration by monocytic-lineage cells expressing *Arg1* and *Vegfa* (Fig. 4C, Additional file 6: Table S5). Both IDH-A and ATRX-KO glioma show significantly increased expression of *FYN* (Additional file 3: Table S2, Additional file 6: Table S5). Recently, *FYN* knockdown was shown to significantly decrease glioma infiltration by myeloid-derived suppressive cells and to significantly increase survival, in an immune-dependent fashion. However, a caveat of our study is that, as previously reported (c.f. Fig. 1A of [35]), ATRX-KO tumors were slower growing. The relative contributions of direct (e.g., cytokine elaboration) vs. indirect (e.g., cell metabolism and growth rates, the epistatic effect of p53 mutations) to the differences in the tumor microenvironment are yet to be fully determined.

Studies of ATRX function in the developing cortex provide a roadmap. ATRX loss alters nucleosome positioning at CCCTC-motif sequences and decreases CTCF binding, this leads to changes in chromatin conformation and gene expression [24]. In our motif analysis of differential peaks between IDH-A and IDH-O scATAC-seq (Fig. 2C, D, Additional file 1: Fig. S2C), we found that CTCF and its paralog CTCFL were the two most significantly over-represented in IDH-O cells, at frequencies eight to nine-fold higher than expected by random chance (Fig. 2D, Additional file 5: Table S4). Likewise, in the scATAC-seq of murine IDH-mutant gliomas, we also observed significant enrichment for CTCF and CTCFL motifs in the peaks that were specific to ATRX-wildtype tumors, but we found no enrichment in ATRX-KO peaks (Fig. 4I, Additional file 7: Table S6). This led us to hypothesize that ATRX KO would lead to a loss of CTCF binding that could explain the global increase in open chromatin as well as the observed changes in gene expression. We performed scCUT&Tag for CTCF on SB28+IDH1R132H ATRX WT and KO cells (Fig. 5, Additional file 1: Fig. S5) to confirm this. Additionally, we integrated our data with published murine NSC chromatin-conformation data [32]. We found that ATRX KO leads to a global decrease in CTCF binding. In particular, we found CTCF loss at NSC chromatin-loop boundaries inferred from Hi-C. We found that gene expression along CTCF-affected loops was correlated, with average gene expression along a given loop significantly increasing or significantly decreasing in a coordinated fashion for loops containing Vegfa, Tnc, Nfia, and others.

## Conclusions

Taken together with ATRX’s known role as a co-factor for CTCF [24], and the known enrichment of ATRX-mediated histone-variant H3.3 at CTCF binding sites [36], we conclude that ATRX deficiency leads to global CTCF loss and the perturbation of chromatin-loop boundaries. These changes in turn induce coordinated loop-wide increases or decreases in gene expression that lead to the observed phenotypes.

## Methods

### Tumor tissue acquisition

We acquired fresh and archival frozen tumor tissue from patients who underwent surgical resection for glioma at UCSF. De-identified samples were provided by the UCSF Neurosurgery Tissue Bank. Sample use was approved by the Institutional Review Board at UCSF. The experiments performed here conform to the principles set out in the WMA Declaration of Helsinki and the Department of Health and Human Services Belmont Report. All patients provided informed written consent.

### Tissue processing for scRNA-seq

Fresh tissues were minced in collection media (Leibovitz’s L-15 medium, 4 mg/mL glucose, 100 u/mL Penicillin, 100 ug/mL Streptomycin) with a scalpel. Samples dissociation was carried out in a mixture of papain (Worthington Biochem. Corp) and 2000 units/mL of DNase I freshly diluted in EBSS and incubated at 37°C for 30 min. After centrifugation (5 min at 300 g), the suspension was resuspended in PBS. Subsequently, suspensions were triturated by pipetting up and down ten times and then passed through a 70-μm strainer cap (BD Falcon). Last, centrifugation was performed for 5 min at 300 g. After resuspension in PBS, pellets were passed through a 40-μm strainer cap (BD Falcon), followed by centrifugation for 5 min at 300 g. The dissociated, single cells were then resuspended in GNS (Neurocult NS-A (Stem Cell Tech.), 2 mM l-glutamine, 100 U/mL penicillin, 100 ug/mL streptomycin, N2/B27 supplement (Invitrogen), sodium pyruvate). Nuclei were extracted from frozen tissues following the “Frakenstein” protocol developed by Luciano Martelotto, Ph.D., Melbourne, Centre for Cancer Research, Victorian Comprehensive Cancer Centre, and available from 10X Genomics (https://community.10xgenomics.com/t5/Customer-Developed-Protocols/ct-p/customer-protocols). Nuclei purification for snRNA-seq was performed by flow cytometry, as described in the Frankenstein protocol. Nuclei purification for scATAC-seq and scCUT&Tag assays was performed via a sucrose-based density gradient: 3200 g for 20 min at 4°C in a 50 ml tube, low-density buffer: 0.32 M sucrose, 10 mM HEPES, 5 mM CaCl2, 3 mM MgAc, 0.1 mM EDTA, and 1 mM DTT in nuclease-free water, high-density buffer: 1 M sucrose, 10 mM HEPES, 3 mM MgAc, and 1 mM DTT.

### Single-cell/nuclei RNA-seq, single-cell ATAC-seq, and single-cell CUT&Tag assays

Tagmentation for scATAC-seq, as well as nuclei capture and library prep for all single-cell assays, was performed as previously described [3], via the 10X Genomics platform. Antibody incubation and tagmentation for scCUT&Tag was done as in [37], with the following modifications. Cell lysis was performed via Nuclei EZ Prep (Sigma) according to the manufacturer’s instructions. Nuclei purification was performed as above via sucrose gradient. Following centrifugation, nuclei were resuspended in 200 ul of Nuclei buffer (10X Genomics) with a 1:50 dilution of primary antibody (anti-CTCF Diagenode C15410210-50) and incubated overnight at 4°C with slow rotation. Nuclei were spun down and resuspended in 200 ul of nuclei buffer with a 1:50 dilution of secondary antibody (Antibodies-online ABIN3042027) and a 1:100 dilution of pA-Tn5 transposase loaded with Illumina barcodes (Diagenode C01070001), then incubated for 1 h at room temperature with slow rotation. Nuclei were counted in a Countess II, then washed and resuspended with sufficient nuclei buffer to achieve a concentration of 2000 nuclei/ul. To perform tagmentation, 5 ul of nuclei suspension was added to 7 ul of ATAC buffer (10X Genomics) and 2 ul of 1:100 loaded pA-Tn5 and incubated at 37°C for 1 h. Nuclei capture and library prep were then performed according to 10X Genomics scATAC-seq protocol. Sequencing was performed according to 10X Genomics recommended protocols on an Illumina NovaSeq 6000.

### Generation of SB28 cell line with ATRX loss and stable IDH1R132H expression

An ATRX CRISPR double-nickase plasmid (Santa Cruz Biotech sc-423734-NIC) was transfected into SB28 mouse glioma cells (a kind gift from Prof. Okada, UCSF) using FuGENE transfection reagent (Promega, Catalog number E2311). The CRISPR guide-RNAs used were CTAACTATGACTCAGAGTTAGAGAGAGAGATAAAAACCATGAGCAGAATT and CTAACTATGACTCAGAGTTAGAGAGAGAGATAAAAACCATGAGCAGAATT target exon 9 of ATRX. Non-targeting guide-RNAs were expressed in controls (Santa Cruz Biotech sc-437281). Following selection via puromycin, single cells were sorted into multi-well plates and expanded as clones. Homozygous knockout was validated by a cleavage assay (Thermofisher, Catalog number: A24372), using primers F: CTACTTCCGGGTCAGATTTTGA, and R: GCACCATCAGAGGAAACACC. Complete loss of ATRX protein was validated by Western blot (Abcam # ab97508). SB28^ATRX-KO^ cells were transfected with plasmids expressing either IDH1R132H (ORIGENE, CAT#: RC400096) or wildtype IDH1 (Origene CAT#: RC210582) and selected via neomycin. Cells were cultured in RPMI media supplemented with 10% FBS and 100 U/mL penicillin and 0.1% streptomycin.

### D2HG assay

Confluent cells were collected and dissociated in CelLytic M (Sigma Catalog Number C2978), several sample dilutions were prepared to ensure the readings were within the range of the standard curve. A perchloric-acid deproteinization protocol was used to remove contaminating proteins from the samples. Standards were prepared in the same extraction buffer used for sample preparation. Black 96-well plates were used for D2HG detection, and 75 μl of D2HG Complete Reaction Mixture (Sigma Catalog Number MAK320) was added into all wells containing samples and standards. The plate was incubated at 37°C for 30–60 min, protected from light. Fluorometric detection was carried out in triplicate with excitation at 540 ± 10 nm and emission of 610 ± 10 nm.

### Cell invasion assay

Cells were trypsinized and plated in serum-free medium in the upper chambers of ECMatrix™-coated polycarbonate membranes (24-well insert, 8 μm pore size—ECM550) (Millipore, Billerica, MA), RPMI supplemented with 10% FBS was added to the lower chambers. Cells were then incubated for an additional 24 h, after which the invasive cells on the lower surface of the membrane were stained, dissociated with 10% acetic acid, and transferred to a 96-well plate. Optical density (OD) was measured at 560 nm.

### Senescence assays

We assayed the activation of β-galactosidase enzyme (β-gal) in SB28 and patient-derived cell lines, as a biomarker for senescent cells in different conditions. ATRX-scrambled-control or ATRX-KO, IDH1R132H OE cells were treated with 4 μM PD0332991(cDK4 inhibitor) or 200 nM Doxorubicin for 4 days. Kit #9860 (Cell Signaling Tech) was used to visualize β-gal expression, according to the manufacturer’s instructions. The CellEvent Senescence Green Detection Kit from Themofisher (Cat. # C10850) was used, according to the manufacturer’s instructions, to quantify β-gal expression in the following treatment. Therapy-induced senescence was likewise assayed in the patient-driven cell lines SF10417 and SF10602 as above, Cell Signaling Technologies kit #9860 was used for visualization and Thermofisher kit # C10850 was used for quantification, with the following modification. Patient-derived cells were seeded and treated with 2 μM PD0332991(cDK4 inhibitor) or 100 nM Doxorubicin or 100 μM of Temozolomide (TMZ) or 2 μM Imatinib for 4 days.

### Animal experiments

All surgical procedures were conducted according to our Institutional Animal Care and Use Committee and NIH ethical guidelines. ATRX-KO and wildtype SB28 cells expressing IDH1R132H and a luciferase reporter were used to establish intracranial tumors in C57BL/6j mice, using 6 mice per cohort. Animals were anesthetized using isoflurane, and each surgical procedure was performed after placing the animal in a stereotactic frame. An aliquot of 5 × 10^4^ SB28 cells was injected into the right parietal region of the brain. The injection site was 2 mm to the right of the central suture and 0.5 mm anterior to the lambda junction; the depth of injection was 2–3 mm. The rate of injection was 1 μl/min. Tumor growth was monitored using in vivo bioluminescence imaging (BLI). For BLI analysis, we defined the region of interest encompassing the intracranial site using Spectral Image analysis software and recorded light emission as the total number of photons per second per steradian per square centimeter. Mice were sacrificed when they showed hunchback, seizures, hemiparesis, or weight loss of greater than 20%; two control mice and one ATRX-KO mouse were found dead and thus excluded. Following euthanasia, mice were perfused with PBS, and tumor tissue was harvested and snap-frozen.

### Single-cell/nuclei RNA-seq data processing

We used CellRanger (version 3.0.2) to pre-process the sc/snRNA-seq data from the 10X Genomics platform. Low-quality cells were filtered as described previously [3]. Copy number variants were inferred via CONICSmat [10] with a threshold of *p*=0.001 and a difference in Bayesian Criterion >100. ELSA [11] was used to classify non-neoplastic cell types. Cluster-specific genes were identified via the FindMarkers/FindAllMarkers function from Seurat package [38], with “MAST” selected as the test-statistic parameter. ScRNA-seq expression data from the Smart-seq platform, in the form of processed expression tables, were downloaded from GSE70630 and GSE89567. Differential gene expression analysis for the Smart-seq data was performed by using limma package [39], as those data are only available publicly as normalized expression tables. Differential expression for the 10X Genomics-based sc/snRNA-seq data was performed via MAST [40]. *P* values for all differential tests were adjusted to control for multiple hypothesis testing via fdrtool [41]. Over-representation of known transcription-factor binding sites, shown in Additional file 1: Fig. S3C, were computed via BART [42]. Lists of known ligand-agonist pairs, used for Fig. 3, were obtained from the Database of Interacting Proteins [43].

### ScATAC-seq and scCUT&Tag data processing and analysis

CellRanger ATAC (version 1.1.0) was used for alignment, deduplication, and identifying transposase cut sites, using the Genome Reference Consortium assembly GRCh38 (hg38) and GRCm38 (mm10) as references for human and murine samples, respectively. The output matrix of CellRanger was further filtered for quality based on two criteria: (1) number of fragments > 1000 and (2) fragments in promoter ratio > 0.2. CONICSmat was used to estimate copy-number variants based on gene-body activity computed via snapATAC [12]. The PCA in Fig. 1C was performed using the cell-by-bin count matrix as input. The clusters shown in Fig. 1C were constructed via the k-nearest neighbor algorithm, as implemented in snapATAC. Peaks and differential peak calls were done in MACS [44] via the “runMACSForAll” and “findDAR” routines in snapATAC. Associations between peaks and nearby genes, used in Additional file 1: Fig. S3A-B and Additional file 1: Fig. S4, were made based on assigning peaks to the nearest gene within 25 Kbp via bedtools [45]. Over-represented transcription-factor motifs were identified both in HOMER (homer.ucsd.edu) via snapATAC’s “runHomer” package and via chromVAR [46]. ChromVAR was likewise used to compute significant changes in motif frequencies between IDH-A and IDH-O cells and to compute motif deviation-from-expected *z* scores. Heatmaps of differential peaks were created in deepTools 3.4.0 [47]. Chromatin-loop calls and associated boundary domains, identified in murine neural stem cells (mNSCs) via Hi-C, were obtained from Pekowska et al. [32] and mapped from 9 to 10mm via liftOver from the UCSC Genome Browser [48]. ATRX binding sites in p53-null mNSCs identified via ChIP-seq were obtained from Danussi et al. [49]. ATRX-KO/WT cell-averaged log fold-change in gene expression, computed via MAST on tumor-derived neoplastic cells as above, was averaged across genes lying within each chromatin loop identified in Pekowska et al. As a control, we generated a background distribution for all loops of a given size (in terms of the number of genes it contained) and chromosome. We included in this control distribution values obtained via averaging over a sliding window of the same size as the loop (number of genes) and ranging across the same chromosome. KEGG pathway enrichment analysis was performed via WebGestalt [50].

## Supplementary Information

**Additional file 1:.** Supplementary Figures S1-S5

**Additional file 2:.** Table S1: Summary of patient and sample molecular characteristics for specimens used in the study.

**Additional file 3:.** Table S2: Differential expression tests between IDH-A and IDH-O cells by cell type, computed via limma.

**Additional file 4:.** Table S3: Summary of markers used and associated references for Fig. 1E.

**Additional file 5:.** Table S4: Differentially enriched motifs and motif enrichment on differential peaks, computed via chromVAR and HOMER respectively, between IDH-A and IDH-O cells by cell type.

**Additional file 6:.** Table S5: Differential expression tests between ATRX-wildtype and ATRX-KO cells in an IDH1R132H background from in vivo tumors by cell type, computed via MAST.

**Additional file 7:.** Table S6: Motif enrichment on differential peaks between ATRX-wildtype and ATRX-KO cells in an IDH1R132H background from in vivo tumors by cell type, computed via HOMER.

**Additional file 8:.** Table S7: NSC chromatin-loops annotated with genomic coordinates, ATRX-WT/KO differential anti-CTCF scCUT&Tag peaks, loop-averaged ATRX-KO/WT cell-averaged in vivo log fold-change in expression in neoplastic cells represented as a percentage of a data-driven null distribution, and the number of genes on each loop.

**Additional file 9:.** Review history

## Data Availability

The raw study data are available from the European Genome-phenome Archive repository under EGAS00001004523 [51]. Processed data is also available from the Gene Expression Omnibus under GSE155430 [52].
