# Supplementary material for: ATRX regulates glial identity and the tumor microenvironment in IDH-mutant glioma
Source: Genome Biol. 2021 Nov 11;22:311. doi: 10.1186/s13059-021-02535-4 (PMC8588616; doi:10.1186/s13059-021-02535-4)
Supplement: Supplementary file 1 — Additional file 1:. Supplementary Figures S1-S5 [file 13059_2021_2535_MOESM1_ESM.pdf]

Supplementary Figures:

Fig. S1: A-C) ScATAC-seq quality-control metrics for the data in this study compared to Ziffra et al. [51]. D) Percentages of neoplastic cells in each of the three clusters found in Fig. 1C. E-F) Transcription-factor motif frequencies in scATAC-seq data for select motifs that are differentially enriched between IDH-A and IDH-O neoplastic cells at q<0.05 (Table 3), assessed via ChromVAR and represented as standardized deviances from expected (Methods) and separated by cluster. Error bars represent standard error. G) Immunohistochemistry for select transcription factors in human cortical specimens, 15 weeks post conception. Images were obtained from the Ivy Glioblastoma Atlas.

Fig. S2: A-B) Transcription-factor motif frequencies in scATAC-seq data for select motifs that are differentially enriched between IDH-A and IDH-O neoplastic cells (Table 3), represented as standardized deviances from expected (Methods) and separated by cluster. Error bars represent standard error. C) Overlap between differentially expressed genes in scRNA-seq and genes within 25 Kbp of an IDH-A/O scATAC-seq differential peak, comparing IDH-A and IDH-O neoplastic cells.

Fig. S3: A-B) Overlaps between differentially expressed genes in scRNA-seq and genes within 25 Kbp of a differential scATAC-seq peak. Overlapping genes, enriched in IDH-A over IDH-O were further restricted to identify transcription factors A) and agonists for monocytic-lineage cell-expressed ligands B). C) Transcription factors whose known binding sites are over-represented in IDH-A-specific peaks near agonist genes, computed via BART.

Fig. S4: A-B) Overlap between differentially expressed genes in snRNA-seq and genes within 25 Kbp of a differential peak in scATAC-seq data, comparing neoplastic cells from ATRX-KO to ATRX-wildtype tumors in an IDH1R132H background. C) Clustering of open-chromatin profiles from scATAC-seq of SB28+IDH1R132H in vivo. D) Motif frequencies via ChromVAR, represented as standardized deviances from a data-driven null distribution, by cluster, comparing ATRX KO with WT neoplastic cells.

Fig. S5: A) Fraction of reads in peak for the scCUT&Tag data. B) As in Fig. 5A, read densities around JASPAR CTCF motifs, except for an IDH-wildtype, ATRX-wildtype glioblastoma tissue specimen used as a positive control. C) KEGG pathway enrichment of genes within 25Kbp of CTCF peaks lost upon ATRX KO, compared to KEGG pathway enrichment of ATRX-KO/WT differential gene expression. D-H) As in Fig. 5E, browser shots, loop-expression percentile and

null distribution, and gene differential expression test and violin plot, for NFKB1 and AUTS2. K) Expression of β-galactosidase in untreated negative controls.

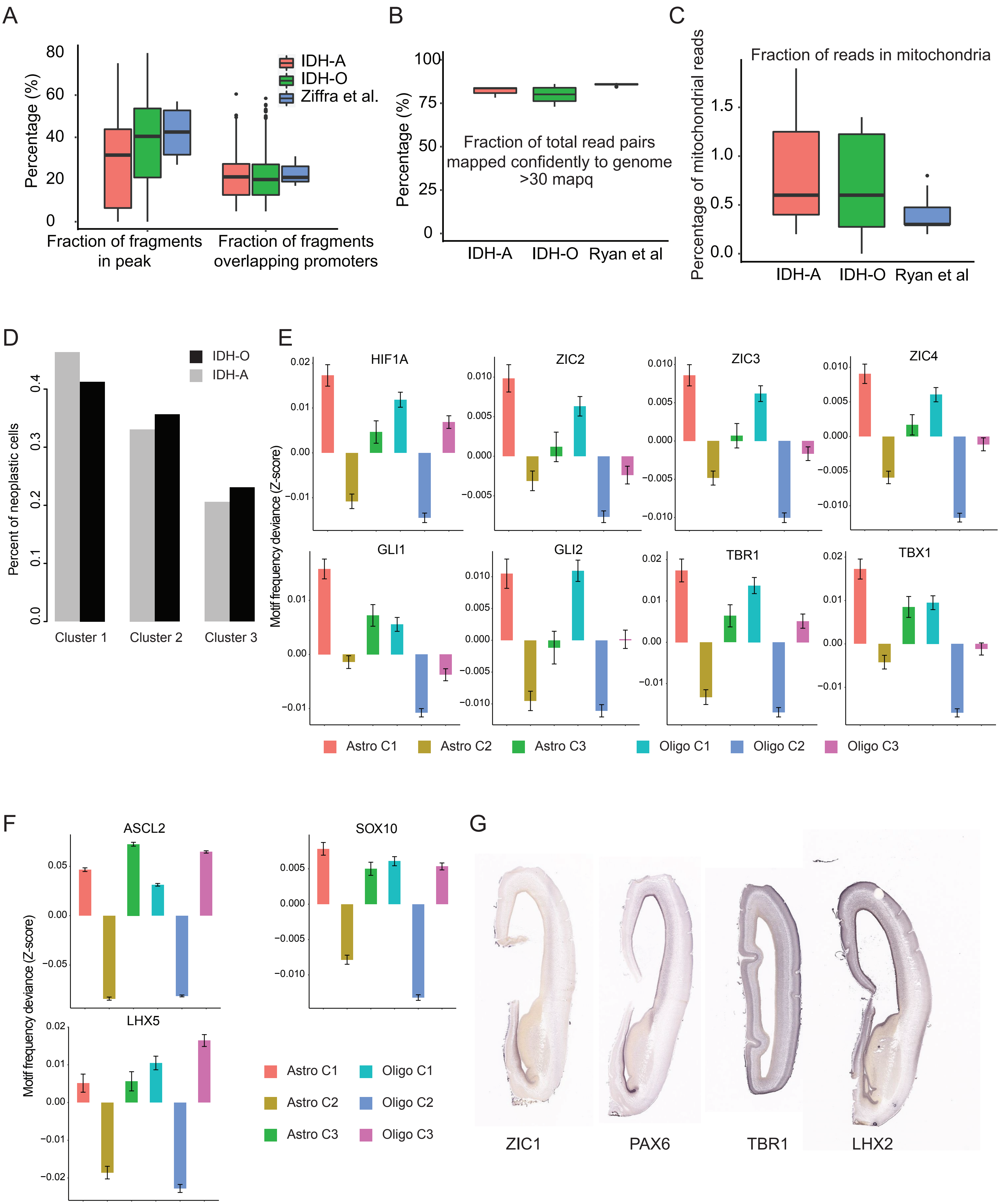
A
Percentage (%)
IDH-A
IDH-O
Ziffra et al.
Fraction of fragments in peak
Fraction of fragments overlapping promoters
B
Percentage (%)
Fraction of total read pairs mapped confidently to genome >30 mapq
IDH-A
IDH-O
Ryan et al
C
Percentage of mitochondrial reads
Fraction of reads in mitochondria
IDH-A
IDH-O
Ryan et al
D
Percent of neoplastic cells
IDH-O
IDH-A
Cluster 1
Cluster 2
Cluster 3
E
Motif frequency deviance (Z-score)
HIF1A
ZIC2
ZIC3
ZIC4
GLI1
GLI2
TBR1
TBX1
Astro C1
Astro C2
Astro C3
Oligo C1
Oligo C2
Oligo C3
F
Motif frequency deviance (Z-score)
ASCL2
SOX10
LHX5
G
ZIC1
PAX6
TBR1
LHX2

Fig S1

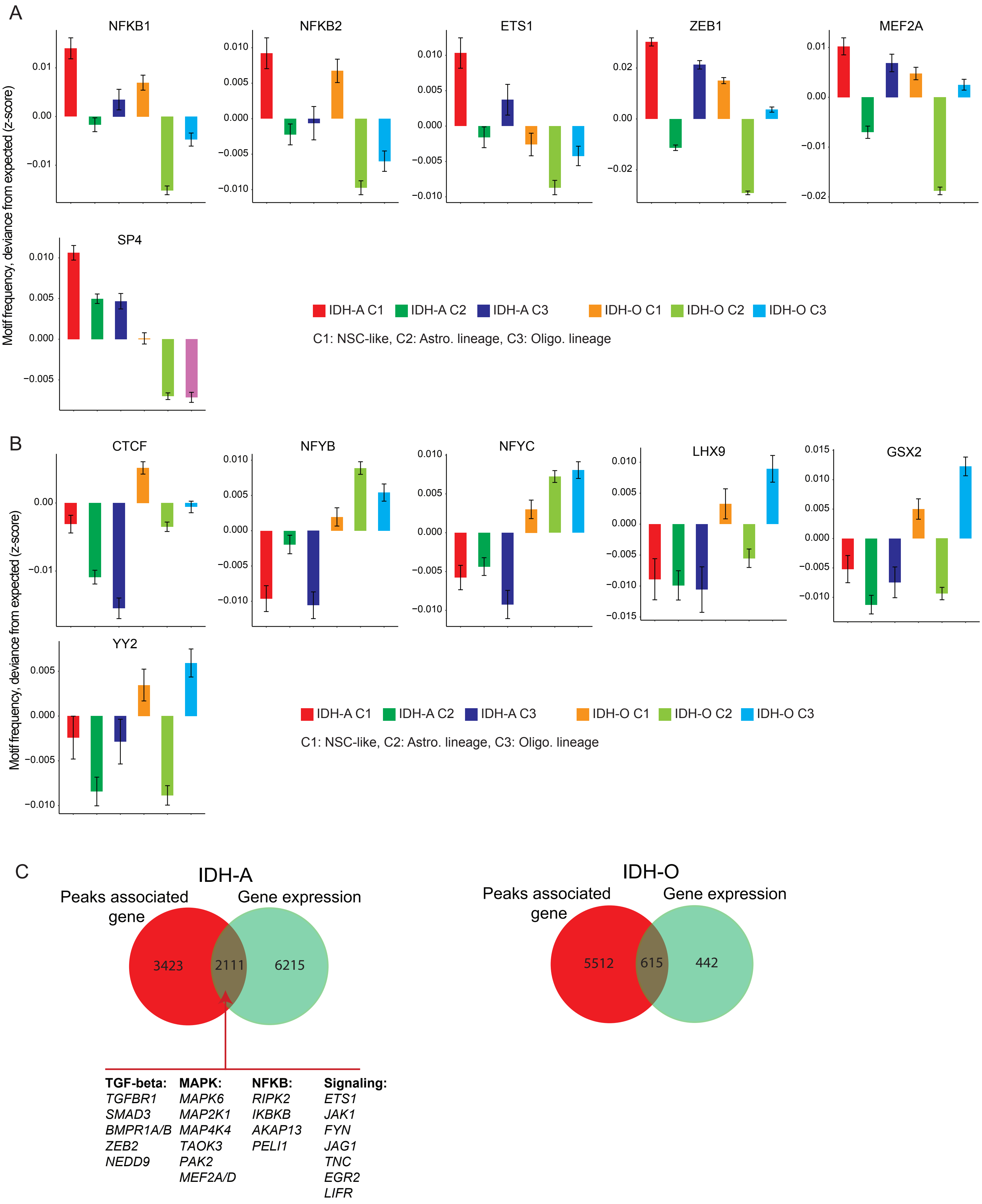
A
NFKB1
NFKB2
ETS1
ZEB1
MEF2A
SP4
Motif frequency, deviance from expected (z-score)
IDH-A C1 IDH-A C2 IDH-A C3 IDH-O C1 IDH-O C2 IDH-O C3
C1: NSC-like, C2: Astro. lineage, C3: Oligo. lineage
B
CTCF
NFYB
NFYC
LHX9
GSX2
YY2
Motif frequency, deviance from expected (z-score)
IDH-A C1 IDH-A C2 IDH-A C3 IDH-O C1 IDH-O C2 IDH-O C3
C1: NSC-like, C2: Astro. lineage, C3: Oligo. lineage
C
IDH-A
Peaks associated gene
Gene expression
3423
2111
6215
IDH-O
Peaks associated gene
Gene expression
5512
615
442
TGF-beta: TGFBR1 SMAD3 BMPR1A/B ZEB2 NEDD9
MAPK: MAPK6 MAP2K1 MAP4K4 TAOK3 PAK2 MEF2A/D
NFKB: RIPK2 IKBKB AKAP13 PELI1
Signaling: ETS1 JAK1 FYN JAG1 TNC EGR2 LIFR

Fig S2

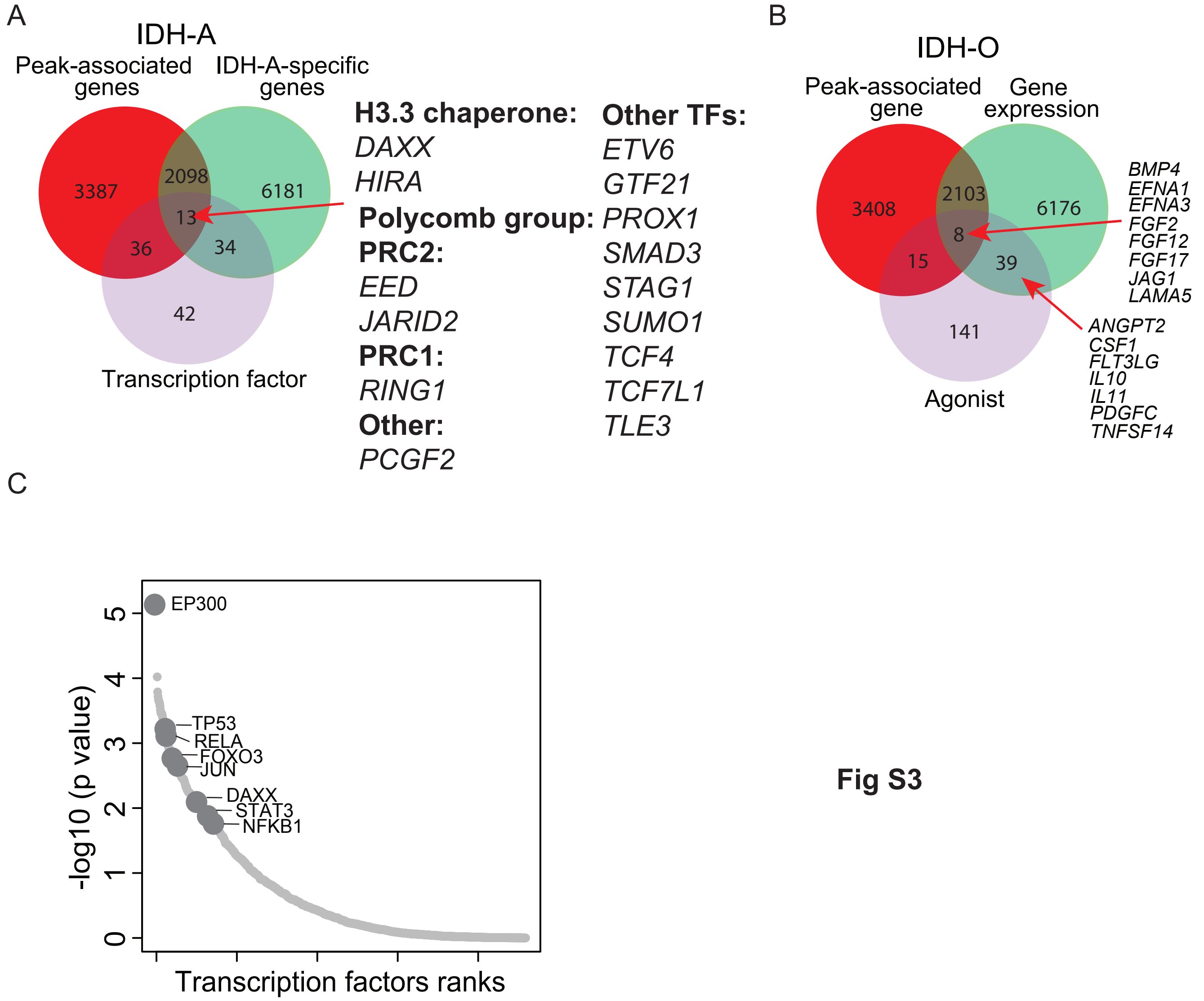
A
IDH-A
Peak-associated genes
IDH-A-specific genes
3387
2098
6181
13
36
34
42
Transcription factor
H3.3 chaperone:
DAXX
HIRA
Polycomb group:
PRC2:
EED
JARID2
PRC1:
RING1
Other:
PCGF2
Other TFs:
ETV6
GTF21
PROX1
SMAD3
STAG1
SUMO1
TCF4
TCF7L1
TLE3
B
IDH-O
Peak-associated gene
Gene expression
3408
2103
6176
8
15
39
141
Agonist
BMP4
EFNA1
EFNA3
FGF2
FGF12
FGF17
JAG1
LAMA5
ANGPT2
CSF1
FLT3LG
IL10
IL11
PDGFC
TNFSF14
C
EP300
TP53
RELA
FOXO3
JUN
DAXX
STAT3
NFKB1
-log10 (p value)
Transcription factors ranks

Fig S3

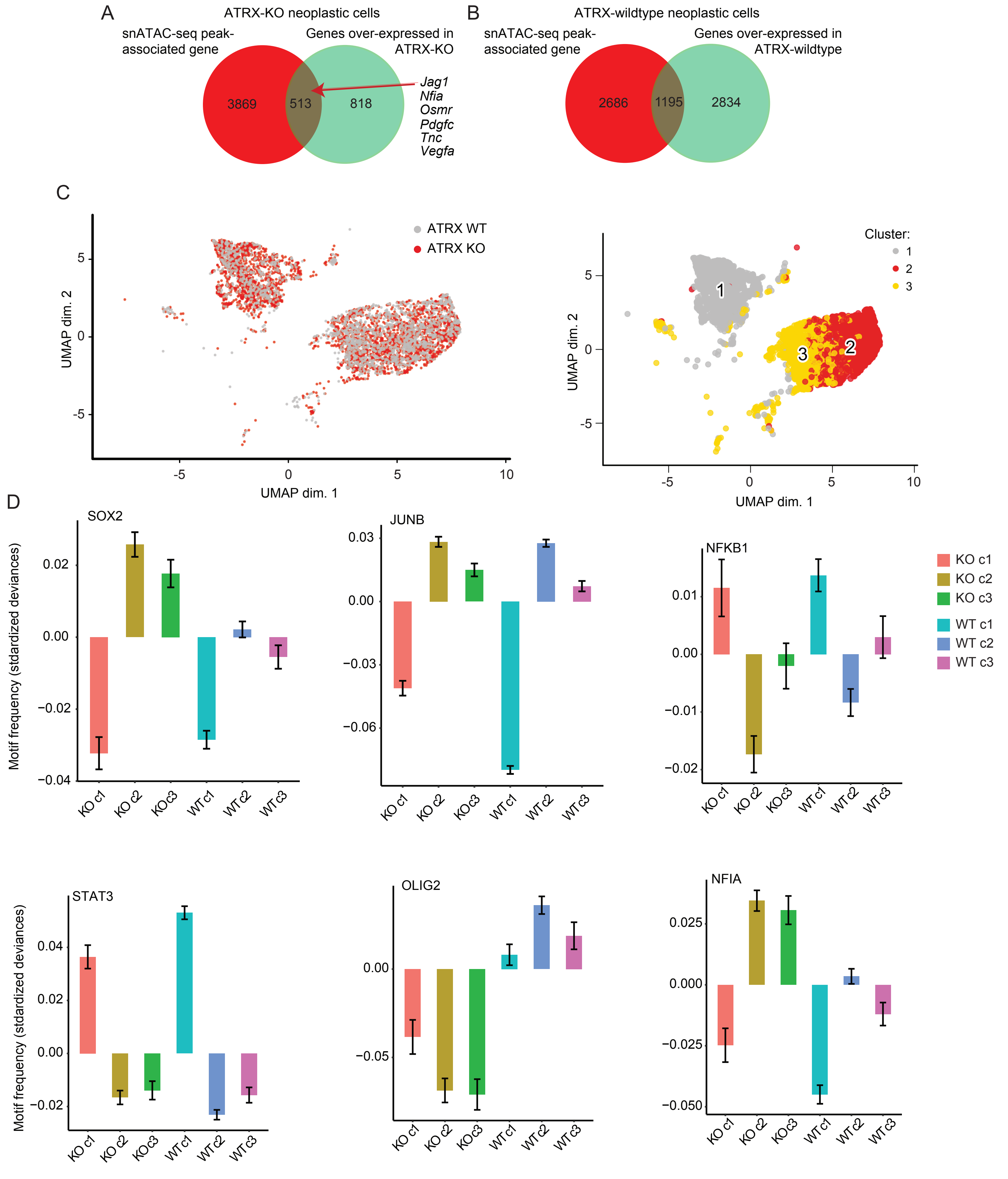
A
ATRX-KO neoplastic cells
snATAC-seq peak-associated gene
Genes over-expressed in ATRX-KO
3869
513
818
Jag1
Nfia
Osmr
Pdgfc
Tnc
Vegfa
B
ATRX-wildtype neoplastic cells
snATAC-seq peak-associated gene
Genes over-expressed in ATRX-wildtype
2686
1195
2834
C
ATRX WT
ATRX KO
UMAP dim. 1
UMAP dim. 2
Cluster:
1
2
3
D
SOX2
JUNB
NFKB1
STAT3
OLIG2
NFIA
Motif frequency (stdardized deviances)
KO c1
KO c2
KO c3
WT c1
WT c2
WT c3

Fig S4

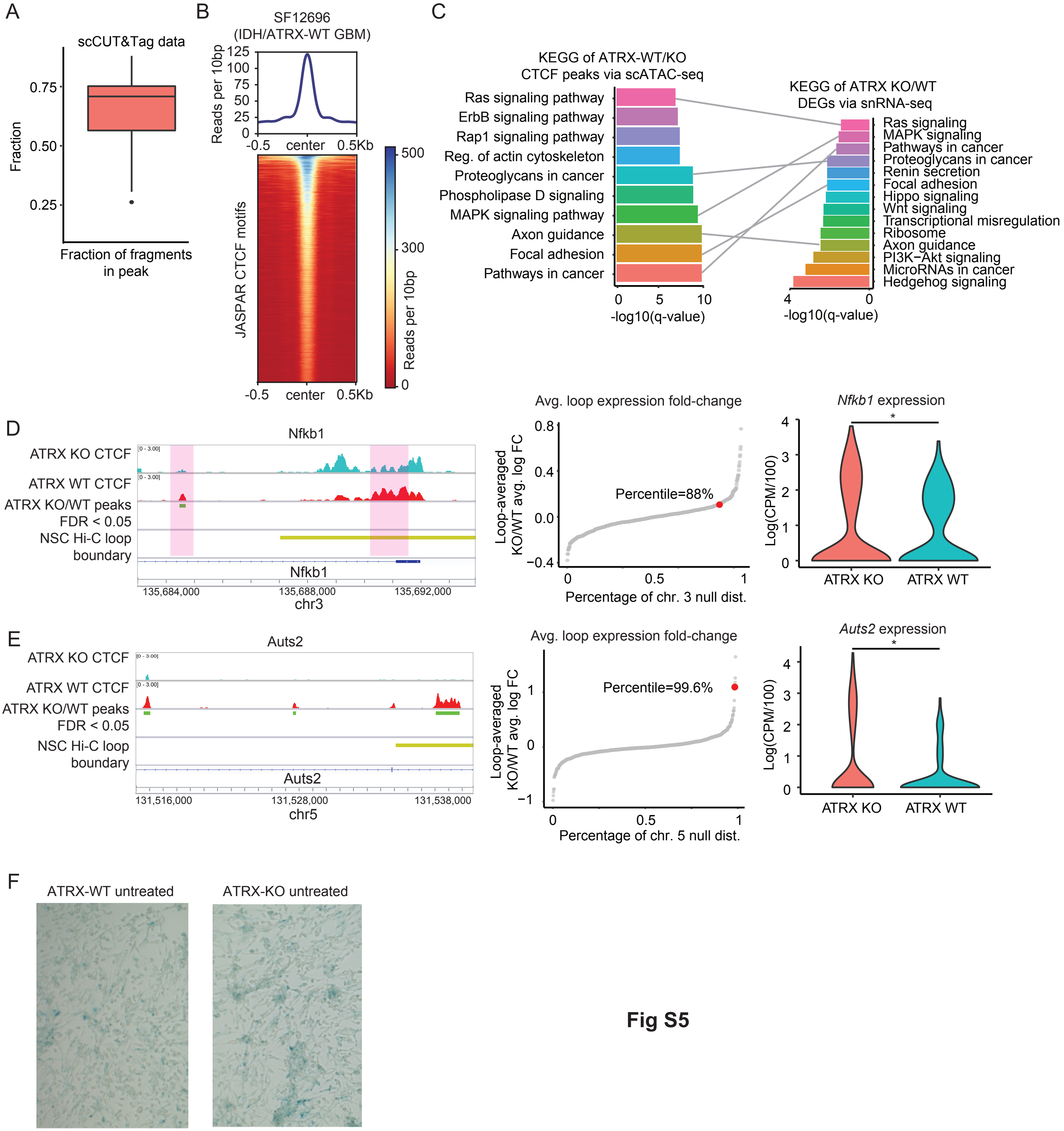
A
scCUT&Tag data
Fraction
0.75
0.50
0.25
Fraction of fragments in peak
B
SF12696 (IDH/ATRX-WT GBM)
Reads per 10bp
125
100
75
50
25
0
-0.5
center
0.5Kb
JASPAR CTCF motifs
500
300
0
Reads per 10bp
C
KEGG of ATRX-WT/KO CTCF peaks via scATAC-seq
Ras signaling pathway
ErbB signaling pathway
Rap1 signaling pathway
Reg. of actin cytoskeleton
Proteoglycans in cancer
Phospholipase D signaling
MAPK signaling pathway
Axon guidance
Focal adhesion
Pathways in cancer
-log10(q-value)
KEGG of ATRX KO/WT DEGs via snRNA-seq
Ras signaling
MAPK signaling
Pathways in cancer
Proteoglycans in cancer
Renin secretion
Focal adhesion
Hippo signaling
Wnt signaling
Transcriptional misregulation
Ribosome
Axon guidance
PI3K−Akt signaling
MicroRNAs in cancer
Hedgehog signaling
-log10(q-value)
D
Nfkb1
ATRX KO CTCF
ATRX WT CTCF
ATRX KO/WT peaks FDR < 0.05
NSC Hi-C loop boundary
135,684,000
135,688,000
135,692,000
chr3
Avg. loop expression fold-change
Loop-averaged KO/WT avg. log FC
Percentile=88%
Percentage of chr. 3 null dist.
Nfkb1 expression
Log(CPM/100)
ATRX KO
ATRX WT
E
Auts2
ATRX KO CTCF
ATRX WT CTCF
ATRX KO/WT peaks FDR < 0.05
NSC Hi-C loop boundary
131,516,000
131,528,000
131,538,000
chr5
Avg. loop expression fold-change
Loop-averaged KO/WT avg. log FC
Percentile=99.6%
Percentage of chr. 5 null dist.
Auts2 expression
Log(CPM/100)
ATRX KO
ATRX WT
F
ATRX-WT untreated
ATRX-KO untreated

Fig S5
