# Supplementary material for: ATRX regulates glial identity and the tumor microenvironment in IDH-mutant glioma
Source: Genome Biol. 2021 Nov 11;22:311. doi: 10.1186/s13059-021-02535-4 (PMC8588616; doi:10.1186/s13059-021-02535-4)
Supplement: Supplementary file 9 — Additional file 9:. Review history [file 13059_2021_2535_MOESM9_ESM.docx]

Review History

**First round of review**

**Reviewer 1**

**Are you able to assess all statistics in the manuscript, including the appropriateness of statistical tests used?**

Yes, and I have assessed the statistics in my report.

**Comments to author:**

This manuscript investigates the epigenetic, transcriptional, and microenvironmental factors promoting IDH-mutant gliomagenesis. Single cell ATAC-seq and RNA-seq are employed to identify changes in chromatin accessibility and downstream transcriptional programs associated with specific molecular abnormalities, along with distinct populations of associated myeloid cells. These approaches are applied to human tumor samples as well as murine models. Particular attention is paid to distinctions between astrocytic gliomas, characterized by ATRX deficiency, and oligodendroglial gliomas with intact ATRX.

The authors recapitulate many earlier findings from other groups regarding the developmental axes upon which IDH gliomas are hierarchically arranged, along with the induction of an astrocytic differentiation profile and increased cellular motility in the context of ATRX deficiency. They also implicate tumor promoting myeloid cells in IDH-mutant astrocytic gliomas. Potential underlying molecular mechanisms related to ATRX deficiency and reduced CTCF functionality are voiced, also with reference to prior work.

The single cell approaches incorporated in this study are innovative and, while many of the core findings described here recapitulate prior literature, they do provide a more thorough thematic synthesis of the factors promoting IDH-mutant and ATRX-mutant gliomagenesis. My comments are below.

1)	The authors ascribe many transcriptional and phenotypic consequences to ATRX deficiency. They should confirm at least a subset of these by re-expressing ATRX in their ATRXKO models. Moreover, while many candidate genes are discussed as likely mediators of the differentiation, migration, and senescence phenotypes described herein, no functional analysis has been performed to confirm these conjectures.

2)	The authors should do more to clarify the mechanism(s) by which ATRX deficiency promotes the observed transcriptional shifts. Prior work (Danussi et al., Nature Comm 2018) linked changes in chromatin accessibility emerging with ATRX deficiency to regions of the genome that largely overlap with ATRX binding sites. Were similar correlations seen here? These data (ATRX ChIP-seq in murine cells) should be publicly available.

3)	Related to point 2, have the authors confirmed changes in CTCF binding patterns by ChIP-seq?

4)	Is the impact of ATRX deficiency on developmental transcription factors and myeloid cells dependent on the co-occurrence of IDH mutation? Were similar findings observed in the absence of IDH mutation? As a corollary to this, the reported 3-fold increase in 2HG expression induced in the IDH-mutant mouse model employed here is much less than that typically seen in the human context (10-100 fold). Are the authors convinced that the biological consequences of IDH mutation are operative in their models? Have they observed the expected DNA and histone methylation changes?

5)	With the availability of extensive single-cell data, the authors seem to have missed an opportunity here to thoroughly investigate the extent to which IDH mutation and ATRX deficiency impact the global epigenome heterogeneously. For instance, what is the variability by which ATRX deficiency impact chromatin accessibility? Are specific changes less robust across the entire sequenced population?

**Reviewer 2**

**Are you able to assess all statistics in the manuscript, including the appropriateness of statistical tests used?**

Yes, and I have assessed the statistics in my report.

**Comments to author:**

This manuscript represents an important dataset for the community (especially for the scATACseq data generated in IDH-mutant gliomas). Unfortunately, many of the interpretations are preliminary and lack the rigor that is required to support the conclusions.

A few points (non-exhaustive) :

-	The abstract/intro suggests that there is a "paradox to solve": that IDH-O and IDH-A have been shown to share lineages, yet differ in genetic alterations and outcome; it is hard to see what paradox there is; differences in genetic alterations (in the same lineage) seem enough of a rationale that can drive differences in clinical behavior; as such, the current understanding in the field is pretty coherent and the question asked by the authors is ill-posed from the onset;

-	The quality of data is not discussed. There is no figure provided to show the quality of the data, which is the foundation of all the analysis. The authors should include the information for some QC analysis for both scRNA-seq and scATAC-seq. E.g. how many reads for every single cell, how many genes captured for every single cell, and what fraction of reads are in peaks for single-cell ATAC-seq. It has been known that the gene expression profile of AC and OC lineages is quite distinct from each other. However, they were clustered together in scATAC-seq data. Is this because the quality of data is suboptimal to capture those differences?

-	the authors identified different transcription factors between IDHA and IDHO via scATAC-seq and found NFI genes dominate IDHA, while OLIG2 and ASCL1 dominate IDHO. Then, the authors found that there are more monocytic-lineage cells from blood in IDHA. Both of these observations are known and have been reported in some prior studies studies (e.g. Venteicher et al. 2017; Azzarelli et al. 2018).

-	The proposed link between CTCF/CTCFL motifs and ATRX loss is NOT supported by their data. Essentially, IDH-A and IDH-O have differences in chromatin accessibility, but their data doesn't show that these differences are due to ATRX loss (e.g. CTCF is not part of the murine model signature).

-	Because ATRX mutation is one of the major genetic differences between IDHA and IDHO, the authors hypothesize that ATRX mutation leads to 1) the upregulation and chromatin enrichment of IDHA master regulator -- NFI genes and 2) upregulation of chemotactic for monocytic-lineage cells. This hypothesis is further investigated in their ATRX KO IDH mutant mouse model. This is the major novel part of the paper but the mechanism of how ATRX contributes to these phenotypes remains unknown and unexplored. E.g. ATRX is known as a chromatin remodeling protein whose main function is the deposition of the histone variant H3.3 to maintain the H3K9me3 modification at heterochromatin. Can the authors do H3.3 and H3K9me3 ChIP-seq in their mouse model to see how ATRX mutant affects the distribution of H3.3 and H3K9me3? This could help us understand why ATRX mutation leads to activation of NFI genes and ligands for monocytic-lineage cells.

-	It should be noted that the human data compared IDH-A to IDH-O (1p/19q codel), but the mouse model uses an artificial glioma model (SB28), then engineers it to be ATRX KO or WT and OE IDHmut; this is of course NOT a great model for the human condition and CERTAINLY not for IDH-O vs IDH-A. The evidence of the representation of the mouse model for the human tumors is not convincing at all. The authors have mentioned that many of these differences between ATRX mutant and WT mouse model agreed with the differences observed between IDH-A and IDH-O human tumors. According to the previous publication (Venteicher et al. 2017), the major expression difference between IDHA and IDHO are attributes to genetics (TP53, ATRX). Can the authors identify the differentially expressed genes between ATRX WT and ATRX KO, and compare to differentially expressed genes between IDHA and IDHO to see if they are enriched in similar pathways? The authors only mentioned a few genes as examples instead of global enrichment analysis.

-	For figure 1E, the authors tried to quantify the programs of different cell types in the NSC-like cells but the analysis result is hard to interpret. The annotation in the figure legend is also not clear. Can the author compare the transcription profile of different cell types in IDH tumors to different cell types in the brain in a pairwise way? So we have a better understanding of their relationships.

-	The experiments on senescence are superficial and add little ATRX KO leads to the ALT (alternative lengthening of telomeres) phenotype which is likely involved in preventing senescence; this is not novel, is not mechanistically dissected and adds little to the existing literature.

Minor questions:

1,Fig2b, y-axis is missing and NKX6-2 seems not expressed in gliomas. Can the author double-check the expression of TFs that they identified to make sure they only present the meaningful ones?

2,Manuscript page 9, line 47, Fig 4H is not consistent with what they described.

Invasiveness experiments in in vitro are unreliable, and difference in in vivo behavior should be shown.

Statistics/significance are frequently not discussed in relevant comparisons

**Authors Response**

**Point-by-point responses to the reviewers’ comments:**

*We thank the Reviewers for the constructive feedback that has helped to improve the manuscript. In the revised manuscript, we have sought to further elucidate the connection between ATRX KO and the gene-expression and phenotypic changes observed in our original report. In particular, we performed single-cell CUT&Tag for CTCF on SB28+IDH1R132H ATRX WT and KO cells (Figures 5, S5). Additionally, we integrated neural stem-cell (NSC) Hi-C data obtained from Pekowska Cell Systems 2018. We found that ATRX KO leads to a global decrease in CTCF binding. In particular, we found CTCF loss at NSC chromatin-loop boundaries inferred from Hi-C. We found that gene expression along CTCF-affected loops was correlated, with average gene expression along a given loop significantly increasing or significantly decreasing in a coordinated fashion for loops containing Vegfa, Tnc, Nfia and others. Taken together with ATRX’s known role as a co-factor for CTCF (e.g. Kernohan Nucleic Acids Res 2014), and the known enrichment of ATRX-mediated histone-variant H3.3 at CTCF binding sites (e.g. Jin Nature Genetics 2009), we conclude that ATRX loss leads to global CTCF loss and the perturbation of chromatin-loop boundaries. These changes in turn induce coordinated loop-wide increases or decreases in gene expression which lead to the observed phenotypes. We hope that the reviewers will agree that these novel findings, when taken together with our global analysis of IDH-mutant clinical specimens, represent a significant advance over the status quo.*

Reviewer #1: This manuscript investigates the epigenetic, transcriptional, and microenvironmental factors promoting IDH-mutant gliomagenesis. Single cell ATAC-seq and RNA-seq are employed to identify changes in chromatin accessibility and downstream transcriptional programs associated with specific molecular abnormalities, along with distinct populations of associated myeloid cells. These approaches are applied to human tumor samples as well as murine models. Particular attention is paid to distinctions between astrocytic gliomas, characterized by ATRX deficiency, and oligodendroglial gliomas with intact ATRX.

The authors recapitulate many earlier findings from other groups regarding the developmental axes upon which IDH gliomas are hierarchically arranged, along with the induction of an astrocytic differentiation profile and increased cellular motility in the context of ATRX deficiency. They also implicate tumor promoting myeloid cells in IDH-mutant astrocytic gliomas. Potential underlying molecular mechanisms related to ATRX deficiency and reduced CTCF functionality are voiced, also with reference to prior work.

The single cell approaches incorporated in this study are innovative and, while many of the core findings described here recapitulate prior literature, they do provide a more thorough thematic synthesis of the factors promoting IDH-mutant and ATRX-mutant gliomagenesis. My comments are below.

1) The authors ascribe many transcriptional and phenotypic consequences to ATRX deficiency. They should confirm at least a subset of these by re-expressing ATRX in their ATRXKO models. Moreover, while many candidate genes are discussed as likely mediators of the differentiation, migration, and senescence phenotypes described herein, no functional analysis has been performed to confirm these conjectures.

*Prior reports have shown that the CTCF loss of binding and associated chromatin-conformation and gene-expression changes, which result from ATRX loss-of-function, are permanent and not rescued upon ATRX restoration (Kernohan Nucleic Acids Res 2014). The phenotypes observed upon ATRX KO should not be reversible by ATRX ectopic expression. When addressing this review, we chose to focus on the Reviewer’s points 2)-3), which were echoed by the second Reviewer. Based on our novel studies in response to these inquiries, the gene-expression and microenvironment composition changes observed in our snRNA-seq, the transcription-factor activities observed in our scATAC-seq, the invasion and senescence assays, as well as the changes in open chromatin, can all be explained by the global loss of CTCF binding we observe upon functional KO of ATRX. The global loss of CTCF binding at chromatin loop boundaries we observe explains both the phenotypic changes as well as the inability to form the heterochromatic foci requisite for therapy-induced senescence. We hope that the Reviewer will agree that these novel assays and analyses, when taken together with our global scATAC-seq analysis of clinical specimens, represents a significant advance over the status quo.*

2) The authors should do more to clarify the mechanism(s) by which ATRX deficiency promotes the observed transcriptional shifts. Prior work (Danussi et al., Nature Comm 2018) linked changes in chromatin accessibility emerging with ATRX deficiency to regions of the genome that largely overlap with ATRX binding sites. Were similar correlations seen here? These data (ATRX ChIP-seq in murine cells) should be publicly available.

3) Related to point 2, have the authors confirmed changes in CTCF binding patterns by ChIP-seq?

*Our original hypothesis was that ATRX loss led to a loss in CTCF binding and a global increase in open chromatin. This was based on the differences in open chromatin observed between human IDH-A and IDH-O samples, as well as between ATRX-KO/WT models (Figures 2C, 4H). Additionally, CTCF motifs were significantly over-represented in ATRX-wildtype scATAC-seq sequences compared ATRX-mutant/KO, in both the human and murine data (Figures 2D, 4I). This interpretation is supported by the senescence assays, as the formation of senescence-associated heterochromatic foci is required for senescence and that ability is lost in ATRX-mutant carcinomas (Kovatcheva Nat Comm 2017).*

*To test this hypothesis and address the Reviewer’s important question, we performed single-cell CUT&Tag for CTCF on SB28+IDH1R132H ATRX WT and KO cells (Figures 5, S5). Additionally, we integrated neural stem-cell (NSC) Hi-C data obtained from Pekowska Cell Systems 2018. We found that ATRX KO leads to a global decrease in CTCF binding. In particular, we found CTCF loss at NSC chromatin-loop boundaries inferred from Hi-C. We found that gene expression along CTCF-affected loops was correlated, with average gene expression along a given loop significantly increasing or significantly decreasing in a coordinated fashion for loops containing Vegfa, Tnc, Nfia and others. Taken together with ATRX’s known role as a co-factor for CTCF (e.g. Kernohan Nucleic Acids Res 2014), and the known enrichment of ATRX-mediated histone-variant H3.3 at CTCF binding sites (e.g. Jin Nature Genetics 2009), we conclude that ATRX loss leads to global CTCF loss and the perturbation of chromatin-loop boundaries. These changes in turn induce coordinated loop-wide increases or decreases in gene expression which lead to the observed phenotypes. While additional studies will need to be done to fully answer the Reviewer’s question, we feel that these findings, when taken together with our global analysis of IDH-mutant clinical specimens, represent a significant advance over the status quo.*

4) Is the impact of ATRX deficiency on developmental transcription factors and myeloid cells dependent on the co-occurrence of IDH mutation? Were similar findings observed in the absence of IDH mutation? As a corollary to this, the reported 3-fold increase in 2HG expression induced in the IDH-mutant mouse model employed here is much less than that typically seen in the human context (10-100 fold). Are the authors convinced that the biological consequences of IDH mutation are operative in their models? Have they observed the expected DNA and histone methylation changes?

*The Reviewer raises an intriguing question as to if the IDH-mutation is required for the phenotypes observed upon ATRX KO. Our preliminary analysis addressing this question indicates that ATRX KO in an IDH-wildtype background in SB28 cells also leads to a global loss of CTCF binding (Figure R1). This is consistent with prior studies in the developing brain connecting CTCF, ATRX and H3.3 deposition to chromatin organization and gene expression (e.g. Kernohan Nucleic Acids Res 2014), which were in an IDH-wildtype context. However, in the clinical setting of adult glioma (as the Reviewer undoubtably knows) ATRX mutations occur almost exclusively in IDH-mutant glioma. In the pediatric setting, however, ATRX mutations are common in diffuse midline glioma (DMG), which is IDH-wildtype. We feel the Reviewer’s question is most relevant to the pediatric context. In order to explore that context further, we will need to generate additional reagents. In particular, we will need to introduce the H3K27M mutations common to DMG or knockout ATRX in an existing DMG model. While we have performed the preliminary CTCF CUT&Tag in ATRX-wildtype/IDH-wildtype SB28 that we have shown here in response to the Reviewer, additional work will be required to interpret those data and additional reagents and studies will be required to follow through in a clinically relevant fashion. Thus, while a compelling question we feel it is somewhat outside of the scope of this study.*

*The Reviewer’s point on 2HG expression is well taken. As the Reviewer is likely aware, models of IDH-mutant glioma are limited. Patient-derived cells from IDH-mutant gliomas typically do not grow in culture at all and when they do they can be passaged only a very limited number of times, making in vitro experiments difficult. Xenografts of patient-derived IDH-mutant cells are also essentially impossible to generate. The engineered models of IDH-mutant glioma also have caveats. However, it is our conviction that our sleeping-beauty (SB) transposon-based spontaneous model represents the state of the art. This model was a generous gift from Hideho Okada, MD PhD, Dir. Brain Tumor Immunotherapy at UCSF. His lab used this model to first demonstrate the immunosuppressive effect of IDH1R132H (Kohanbash JCI 2017). In our hands the production of 2HG is comparable to published reports from other labs using the same model. For example, the Castro lab also used the same SB-based model with similar 2HG differentials to first show the role of IDH1R132H in the DNA-damage response (c.f. Figure R2). Lastly, since our focus is on the consequences of ATRX KO we feel that the limitations of the model are somewhat mitigated, since they are present in both the ATRX WT and ATRX KO condition.*

5) With the availability of extensive single-cell data, the authors seem to have missed an opportunity here to thoroughly investigate the extent to which IDH mutation and ATRX deficiency impact the global epigenome heterogeneously. For instance, what is the variability by which ATRX deficiency impact chromatin accessibility? Are specific changes less robust across the entire sequenced population?

*In the revised manuscript, we include in Figure S4 a discussion of the heterogeneity of the snATAC-seq from murine tumors. We found that in both ATRX KO and WT cells open-chromatin signatures in neoplastic cells were divided into three clusters. The first was depleted of the stemness and proliferation TF-motifs SOX2 and JUNB and dominated by TF-motifs of core components of the expression and transduction of pro-inflammatory cytokines, e.g. NFKB and STAT3 signaling (Figure S4C-D). The second and third were more closely aligned with each other, enriched for SOX2 and JUNB but differed in the relative frequencies of glial-lineage TF motifs. We found a relatively homogeneous shift in all populations in the frequencies of glial lineage motifs from oligodendrocyte-lineage markers (e.g. OLIG2) to astrocytic (e.g. NFIA) across all three populations. The impact of ATRX deficiency on the global epigenome appears to be relatively homogeneous in our model. All of our assays have been done in an IDH-mutant background (see our response to question four).*

Reviewer #2: This manuscript represents an important dataset for the community (especially for the scATACseq data generated in IDH-mutant gliomas). Unfortunately, many of the interpretations are preliminary and lack the rigor that is required to support the conclusions.

A few points (non-exhaustive) :

- The abstract/intro suggests that there is a "paradox to solve": that IDH-O and IDH-A have been shown to share lineages, yet differ in genetic alterations and outcome; it is hard to see what paradox there is; differences in genetic alterations (in the same lineage) seem enough of a rationale that can drive differences in clinical behavior; as such, the current understanding in the field is pretty coherent and the question asked by the authors is ill-posed from the onset;

*We apologize if we’ve overstated our point in the abstract. We mean only to convey a sentiment similar to what the Reviewer writes here, that IDH-O and IDH-A share lineages yet differ in genetic alterations, phenotypes, and outcomes.*

- The quality of data is not discussed. There is no figure provided to show the quality of the data, which is the foundation of all the analysis. The authors should include the information for some QC analysis for both scRNA-seq and scATAC-seq. E.g. how many reads for every single cell, how many genes captured for every single cell, and what fraction of reads are in peaks for single-cell ATAC-seq. It has been known that the gene expression profile of AC and OC lineages is quite distinct from each other. However, they were clustered together in scATAC-seq data. Is this because the quality of data is suboptimal to capture those differences?

*We compared our QC metrics to public scATAC-seq data from human brain specimens (Figure S1). We find that in-peak reads and other metrics are on par with published data. We respectfully disagree with the Reviewer’s assessment of the AC/OC separation. The AC and OC lineages are almost completely separated by positive vs. negative loading along the PC2 axis (C2 5.35% pos vs. 94.64% neg, C3 78.9% pos vs. 21.1% neg); also they cluster apart in the hierarchical clustering (Figure 1C). These observations indicate that the AC and OC lineages have very distinct epigenetic profiles. Lastly, the differences between AC and OC lineages the reviewer describes are at the transcriptomic level. To our knowledge, this is the first single-cell ATAC profiling of these lineages in human IDH-mutant gliomas and we can’t assume a priori that the ATAC-based open-chromatin data will show the same result as prior transcriptomics studies.*

- the authors identified different transcription factors between IDHA and IDHO via scATAC-seq and found NFI genes dominate IDHA, while OLIG2 and ASCL1 dominate IDHO. Then, the authors found that there are more monocytic-lineage cells from blood in IDHA. Both of these observations are known and have been reported in some prior studies studies (e.g. Venteicher et al. 2017; Azzarelli et al. 2018).

*We have carefully reviewed both Venteicher 2017 and Azzarelli 2018 and we do not find any mention of nuclear factor IA (NFIA) or other nuclear factor I (NFI) genes in their texts. To our knowledge, ours is the first report to associate NFIA with AC vs. OC lineage commitment in IDH-mutant glioma.*

*Moreover, while Venteicher 2017, Mueller 2017 and others show a correlation between IDH-A and immunosuppressive monocytic-lineage cells compared to IDH-O, to our knowledge we are the first to test this functionally and to associate this change with ATRX loss-of-function. Our finding that immunosuppressive infiltrates increase in vivo upon ATRX KO is novel.*

- The proposed link between CTCF/CTCFL motifs and ATRX loss is NOT supported by their data. Essentially, IDH-A and IDH-O have differences in chromatin accessibility, but their data doesn't show that these differences are due to ATRX loss (e.g. CTCF is not part of the murine model signature).

*With all due respect, CTCF is part of the murine model signature described in the original manuscript (Figure 4I; Table 6, q=0.0197). We highlighted CTCF as part of the murine model signature as the putative source of gene dysregulation in the murine model, in the results (lines 233-240) as well as in the discussion (lines 300-304).*

*In the revised manuscript we expand on this finding. We now show via CUT&Tag that CTCF-DNA binding drops precipitously upon ATRX KO (Figure 5, S5). Lost CTCF peaks coincide with the boundary domains of NSC TADs and ATRX binding sites. Correlated changes in intra-loop gene expression to loop-boundary CTCF perturbation explain our observed ATRX KO/WT differential gene programs and the phenotypes of invasiveness and enhanced immunogenicity.*

- Because ATRX mutation is one of the major genetic differences between IDHA and IDHO, the authors hypothesize that ATRX mutation leads to 1) the upregulation and chromatin enrichment of IDHA master regulator -- NFI genes and 2) upregulation of chemotactic for monocytic-lineage cells. This hypothesis is further investigated in their ATRX KO IDH mutant mouse model. This is the major novel part of the paper but the mechanism of how ATRX contributes to these phenotypes remains unknown and unexplored. E.g. ATRX is known as a chromatin remodeling protein whose main function is the deposition of the histone variant H3.3 to maintain the H3K9me3 modification at heterochromatin. Can the authors do H3.3 and H3K9me3 ChIP-seq in their mouse model to see how ATRX mutant affects the distribution of H3.3 and H3K9me3? This could help us understand why ATRX mutation leads to activation of NFI genes and ligands for monocytic-lineage cells.

*We agree with the reviewer that the observed effect of ATRX loss on glial-lineage and immunoregulatory genes is novel. As the Reviewer points out, the ATRX-DAXX complex chaperones H3.3 histone cores for deposition. Moreover, CTCF co-localizes with H3.3 histones (Jin Nature Genetics 2009). Taken together with the marked increase in open chromatin observed in scATAC-seq of ATRX-KO/mutant specimens, the enrichments for CTCF motifs in scATAC-seq of ATRX-intact specimens in human and in mouse, and the inability to senesce in both human and murine ATRX-mutant cells, we hypothesized that ATRX knockout led to a depletion of CTCF binding and global chromatin de-condensation. Through a comparison of scATAC-seq differential peaks to nearby differentially expressed genes (Figure S4A), we further attributed the changes in Vegfa, Tnc and other genes and associated phenotypes to this mechanism.*

*In the revised manuscript, to answer the Reviewer’s important question, we performed single-cell CUT&Tag for CTCF on SB28+IDH1R132H ATRX WT and KO cells (Figures 5, S5). Additionally, we integrated neural stem-cell (NSC) Hi-C data obtained from Pekowska Cell Systems 2018. We found that ATRX KO leads to a global decrease in CTCF binding. In particular, we found CTCF loss at NSC chromatin-loop boundaries inferred from Hi-C. We found that gene expression along CTCF-affected loops was correlated, with average gene expression along a given loop significantly increasing or significantly decreasing in a coordinated fashion for loops containing Vegfa, Tnc, Nfia and others. Taken together with ATRX’s known role as a co-factor for CTCF (e.g. Kernohan Nucleic Acids Res 2014), and the known enrichment of ATRX-mediated histone-variant H3.3 at CTCF binding sites (e.g. Jin Nature Genetics 2009), we conclude that ATRX loss leads to global CTCF loss and the perturbation of chromatin-loop boundaries. These changes in turn induce coordinated loop-wide increases or decreases in gene expression which lead to the observed phenotypes. Additional studies will need to be done to fully answer the Reviewer’s question. The Reviewer points to H3K9me3 and H3.3 ChIP-seq. We agree that these studies are important, together with additional anti-ATRX ChIP and Hi-C assays. In our opinion, these should be done via scCUT&Tag in clinical specimens and will require additional development of reagents and optimizations. We hope the Reviewer will agree that the findings and data presented here, when taken together with our global scATAC-seq analysis of IDH-mutant clinical specimens, represent a significant advance over the status quo.*

- It should be noted that the human data compared IDH-A to IDH-O (1p/19q codel), but the mouse model uses an artificial glioma model (SB28), then engineers it to be ATRX KO or WT and OE IDHmut; this is of course NOT a great model for the human condition and CERTAINLY not for IDH-O vs IDH-A. The evidence of the representation of the mouse model for the human tumors is not convincing at all. The authors have mentioned that many of these differences between ATRX mutant and WT mouse model agreed with the differences observed between IDH-A and IDH-O human tumors. According to the previous publication (Venteicher et al. 2017), the major expression difference between IDHA and IDHO are attributes to genetics (TP53, ATRX). Can the authors identify the differentially expressed genes between ATRX WT and ATRX KO, and compare to differentially expressed genes between IDHA and IDHO to see if they are enriched in similar pathways? The authors only mentioned a few genes as examples instead of global enrichment analysis.

*Sleeping-beauty (SB) transposon-based spontaneous glioma models that express IDH1R132H, of which ours is an example, are the most faithful immunocompetent models of IDH-mutant glioma available. We obtained our ATRX-WT baseline model as a generous gift from Dr. Hideho Okada, Director of Brain-tumor Immunotherapy UCSF, who used this model to first demonstrate the effect of IDH-mutations on glioma immunogenicity (Kohanbash JCI 2017). These spontaneous-glioma models are also the immunocompetent IDH-mutant glioma model of choice for the Castro (Nunez Sci Trans Med 2019), Holland (Amankulor Genes Dev 2017), Koschmann (Koschmann Sci Trans Med 2016) and other labs, representing some of the world’s leading experts in IDH-mutant glioma. In terms of alternatives, the only other immunocompetent murine model of IDH-mutant glioma, to our knowledge, is based on GL261 cells which are also syngeneic to BL/6 mice (e.g. Bunse 2018, Pellegatta 2015). However, GL261 cells are hypermutated and produce a strong anti-tumor immune and T-cell response when injected into mouse brains that is not observed in human tumors. As the author well knows, IDH-mutant gliomas at initial presentation are typically lower-grade lesions. The spontaneous glioma models that we use produce only a modest inflammatory response. They are completely resistant to immune-checkpoint blockade as in the human disease, for example. Thus, respectfully, we argue that this is the best model to use to study the effect of ATRX loss in IDH-mutant glioma in an immunocompetent setting.*

*The reviewer asks us to compare the global differences between IDH-A and IDH-O with the differences between ATRX-WT and KO SB28+IDH1R132H. However, we do not claim that the differences between the SB28 models recapitulate those between IDH-A and IDH-O. Modeling all of the differences between IDH-A and IDH-O would additionally require modeling the 1p/19q co-deletion and other alterations. Rather, we seek just to elucidate which of the IDH-A/O differences can be explained by ATRX loss. For this we need an engineered model if we want to consider immunological differences in vivo. For the reasons listed above, SB-based spontaneous models are state-of-the-art for our purposes.*

- For figure 1E, the authors tried to quantify the programs of different cell types in the NSC-like cells but the analysis result is hard to interpret. The annotation in the figure legend is also not clear. Can the author compare the transcription profile of different cell types in IDH tumors to different cell types in the brain in a pairwise way? So we have a better understanding of their relationships.

*We have clarified figure 1E along the lines of the author’s feedback, adding a legend of cell-type acronyms. Additionally, we have summarized the bar graphs as word clouds to convey the take-home point more succinctly: while the stem-like cells from both IDH-A and IDH-O tumors do express markers of NSC and glial-progenitors from the developing neocortex, IDH-A stem-like cells express core NSC transcription-factors much more highly. Furthermore, only IDH-A stem-like cells express proneural (i.e. indicative of neurogenic capacity, e.g. NEUROD1) transcription-factors.*

- The experiments on senescence are superficial and add little ATRX KO leads to the ALT (alternative lengthening of telomeres) phenotype which is likely involved in preventing senescence; this is not novel, is not mechanistically dissected and adds little to the existing literature.

*To our knowledge, there is no connection between ALT and therapy-induced senescence described in the literature, and we do not postulate one here. Therapy-induced senescence requires the formation of heterochromatic foci that institute constitutive gene silencing. In carcinomas, ATRX accumulates in senescence-associated heterochromatic foci and ATRX knockdown leads to resistance to therapy-induced senescence (Kovatcheva Nat Comm 2017). Ours is the first report of ATRX KO leading to senescence resistance in IDH-mutant glioma that we are aware of. The inability to senesce is supporting evidence of our hypothesis that ATRX KO leads to a loss of CTCF binding, dysregulated DNA looping, and an inability to properly condense chromatin. Since the senescence assays are novel, provide support for our central hypothesis, and point to a novel potential mechanism for therapy development, we have chosen to retain this figure panel*

**Second round of review**

**Reviewer 1**

This paper is considerably improved, especially with regard to the inclusion of CTCF profiling and downstream mechanistic insights. With regard to my comments, it is disappointing that, in response to my comment #4, more work was not done to flesh out the specific role of IDH mutation in modulating the myeloid microenvironment, as IDH mutation and 2HG have been repeatedly shown to impact this cellular subcompartment. Nevertheless, I don't feel that these deficiencies justify holding up what is otherwise and excellent paper any longer. The authors should be commended for their outstanding work.

**Reviewer 2**

The authors have significantly strengthened their manuscript and adequately addressed my concerns.
